# Evolutionary analyses reveal immune cell receptor GPR84 as a conserved receptor for bacteria-derived molecules

**DOI:** 10.1016/j.isci.2022.105087

**Published:** 2022-09-06

**Authors:** Amadeus Samuel Schulze, Gunnar Kleinau, Rosanna Krakowsky, David Rochmann, Ranajit Das, Catherine L. Worth, Petra Krumbholz, Patrick Scheerer, Claudia Stäubert

**Affiliations:** 1 Rudolf Schönheimer Institute of Biochemistry, Faculty of Medicine, Leipzig University, Johannisallee 30, 04103 Leipzig, Germany; 2 Charité – Universitätsmedizin Berlin, corporate member of Freie Universität Berlin and Humboldt-Universität zu Berlin, Institute of Medical Physics and Biophysics, Group Protein X-ray Crystallography and Signal Transduction, Charitéplatz 1, 10117 Berlin, Germany; 3 Yenepoya Research Centre, Yenepoya University, Mangalore, Karnataka, India; 4 Independent Data Lab UG, Frauenmantelanger 31, 80937 Munich, Germany

**Keywords:** Biological sciences, Evolutionary biology, Evolutionary processes

## Abstract

The G protein-coupled receptor 84 (GPR84) is found in immune cells and its expression is increased under inflammatory conditions. Activation of GPR84 by medium-chain fatty acids results in pro-inflammatory responses. Here, we screened available vertebrate genome data and found that GPR84 is present in vertebrates for more than 500 million years but absent in birds and a pseudogene in bats. Cloning and functional characterization of several mammalian GPR84 orthologs in combination with evolutionary and model-based structural analyses revealed evidence for positive selection of bear GPR84 orthologs. Naturally occurring human GPR84 variants are most frequent in Asian populations causing a loss of function. Further, we identified *cis*- and *trans*-2-decenoic acid, both known to mediate bacterial communication, as evolutionary highly conserved ligands. Our integrated set of approaches contributes to a comprehensive understanding of GPR84 in terms of evolutionary and structural aspects, highlighting GPR84 as a conserved immune cell receptor for bacteria-derived molecules.

## Highlights

• GPR84 is conserved in vertebrates but absent in birds, and a pseudogene in bats
• Signature of diversifying positive selection in bear GPR84 orthologs
• The quorum-sensing molecules *cis*- and *trans*-2-decenoic acid are GPR84 agonists
• GPR84 loss-of-function variants are most frequent in Asian populations

## Graphical abstract

**Figure:**
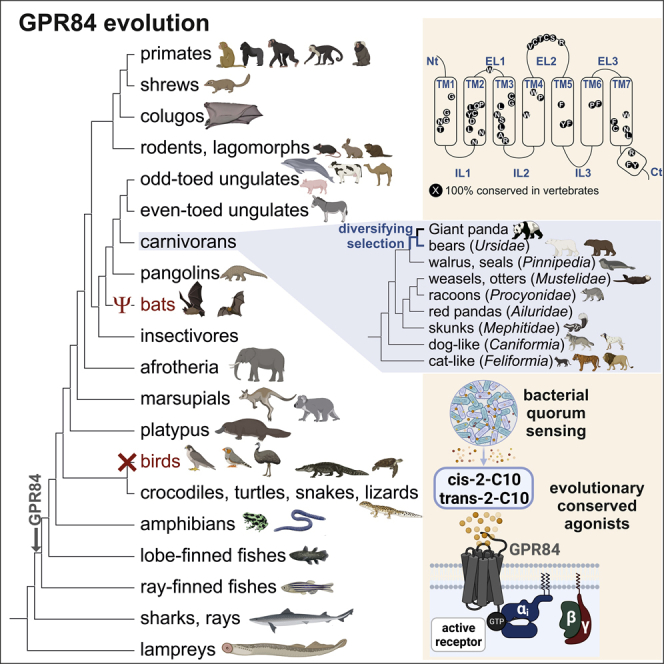

## Introduction

GPR84 is a rhodopsin-like G protein-coupled receptor (GPCR) discovered 20 years ago that is highly expressed in various types of innate immune cells (neutrophils, monocytes, and macrophages) (Wittenberger et al., 2001; Yousefi et al., 2001). We recently demonstrated that this Gα_i_-coupled receptor additionally recruits Gα_15_ proteins in macrophages and neutrophils (Peters et al., 2022). Many studies show that GPR84 expression is increased under pro-inflammatory conditions, including bacterial and viral infections (reviewed in (Luscombe et al., 2020; Marsango et al., 2020; Wojciechowicz and Ma’ayan, 2020)). Activation of GPR84 by medium-chain fatty acids (MCFAs, 9–12 carbons) (Wang et al., 2006) and their respective 3-hydroxy derivatives (3-OH-MCFAs) (Suzuki et al., 2013) results in responses like chemotaxis, phagocytosis, and production of reactive oxygen species (Recio et al., 2018; Lucy et al., 2019; Peters et al., 2022). MCFAs may originate from dietary sources, whereas 3-OH-MCFAs are elevated during ketosis, but are also present in lipopolysaccharides (LPS), which are outer cell-membrane components of gram-negative bacteria (Szponar et al., 2003; Leker et al., 2017; Kutschera et al., 2019). We recently provided direct evidence for the gram-negative bacteria-derived origin of 3-OH-MCFAs (Peters et al., 2022). Besides its role in immune response, several other functions have been reported for GPR84, including regulation of mitochondrial metabolism in skeletal muscle and modulation of fibrotic disease progression (Gagnon et al., 2018; Montgomery et al., 2019). Furthermore, the expression of GPR84 in enteroendocrine ghrelin X/A-like cells indicates that receptor stimulation with MCFAs contributes to the regulation of food intake (Widmayer et al., 2017; Peiris et al., 2021; Velasco et al., 2021). Another recent study showed that GPR84 acts as a gustatory receptor for MCFAs in the oral cavity in mammals, suggesting that MCFAs are relevant for fat taste (Liu et al., 2021). Nevertheless, because of the low concentration of MCFAs and 3-OH-MCFAs *in vivo* combined with their rather modest potency, GPR84 officially remains an orphan receptor (Jenske and Vetter, 2008; Alexander et al., 2021).

From an evolutionary perspective, it is remarkable to point out that no GPR84 paralogs exist in the human genome (Joost and Methner, 2002), although GPR84 is already present in zebrafish and thus appeared more than 400 million years (Myr) ago (Huang et al., 2014).

In the present study, we hypothesized that GPR84 orthologs from diverse mammalian species exhibit variations in sequence and signaling properties as a result of species- or order-specific adaptations to factors like habitat or lifestyle, including diet and associated microbial challenges for the immune system. To address this hypothesis, GPR84 sequences of more than 200 vertebrate species (ortholog identification) were collected from public databases and analyzed for conservation/variation, loss of selective constraints, or signatures of positive selection. Several mammalian GPR84 orthologs were cloned and functionally compared. The selected mammalian species colonize diverse habitats and thus are faced with various environmental conditions, but also differ in size and dietary requirements.

Although several potent surrogate GPR84 ligands have been described (Mahmud et al., 2017; Pillaiyar et al., 2017, 2018; Köse et al., 2020), we stimulated the mammalian GPR84 orthologs with the naturally occurring MCFA decanoic acid (C10) and the 3-OH-MCFA 3-hydroxydecanoic acid (3-OH-C10). Both, C10 and 3-OH-C-10 are assumed to be orthosteric endogenous ligands, and thus their binding sites are more likely subjected to purifying evolutionary selection, whereas allosteric sites are usually more divergent (reviewed in (Wenthur et al., 2014)). In addition, naturally occurring human GPR84 variants, i.e. heterozygous single nucleotide polymorphisms (SNPs) and somatic mutations, were functionally analyzed regarding their impact on GPR84 signaling. These studies were accompanied by GPR84 homology models to extrapolate hot spots of receptor variations in the 3D structure, and thereby associate sequence information with functional data at a structural level. Finally, we tested so far unknown potential GPR84 ligands, considering the role of this receptor as an immune cell receptor. We discovered the bacterial quorum sensing molecules *cis*-2-decenoic acid (*cis*-2-C10) and *trans*-2-decenoic acid (*trans*-2-C10) as potent agonists of mammalian GPR84 orthologs.

Our combined set of approaches yields insights into the evolutionary history of GPR84 as a conserved receptor for microbiota-derived metabolites with relevance for immune function.

## Results

It has previously been shown that GPR84 is present in zebrafish and mediates, as in humans, pro-inflammatory signaling on activation, reflected in enhanced phagocytosis in macrophages (Huang et al., 2014; Recio et al., 2018; Lucy et al., 2019; Wang et al., 2019). Thus, because of its functionality in teleost fish, GPR84 occurrence has been dated back at least 400 Myr (Renshaw and Trede, 2012). Here, we mined publicly available nucleotide databases to collect vertebrate GPR84 ortholog data (NCBI accession numbers in Table S1), which we analyzed for changing evolutionary constraints and sequence conservation. To our best knowledge, no study has so far systematically and functionally addressed aspects of GPR84 vertebrate evolution.

### GPR84 is an evolutionary “old” receptor being already present in jawless fish

**Figure 1:**
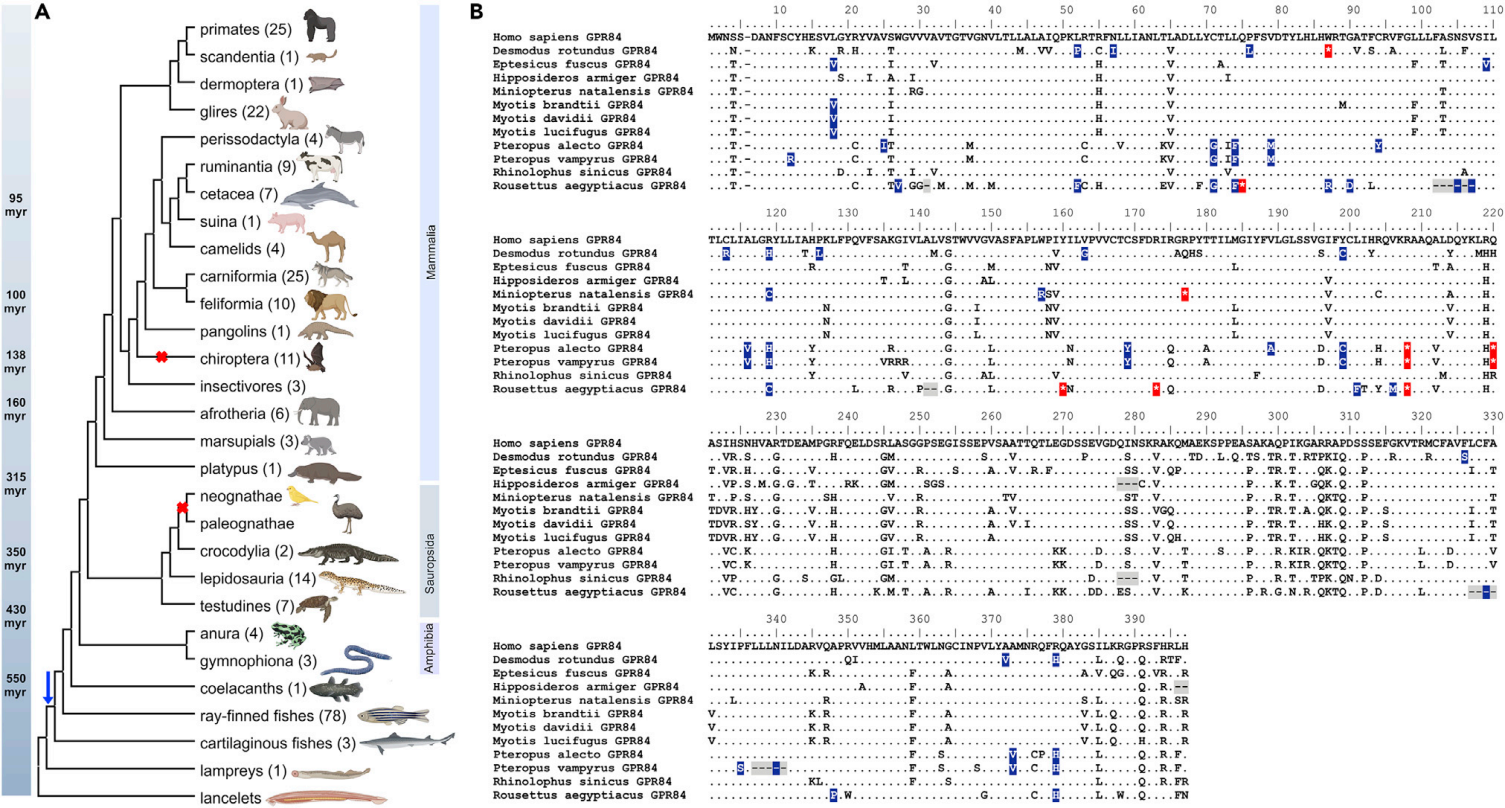
Evolution and pseudogenization of GPR84 in vertebrates (A) Database mining revealed the presence of GPR84 already in lampreys, sharks and rays (cartilaginous fishes). No GPR84 orthologs were found in birds (neognathae and paleognathae). In bats, GPR84 is a pseudogene. The number of species representing each order is indicated in brackets (see also Figures S1, S2, S3, Table S1). Cartoons of species were created with BioRender.com. Divergence times are derived from (Hallström and Janke, 2008; Irisarri et al., 2017). (B) Protein alignment of bat GPR84 orthologs in comparison to human GPR84 to highlight positions reflecting its pseudogenization. Deletions are marked in grey, mutations at otherwise conserved positions in blue, and in-frame stop codons in red.

Our analyses revealed that GPR84 is present in ray-finned fishes, amphibians, turtles, lizards, snakes, crocodilians, and most mammalian orders (Figures 1, S1, S2, Table S1). Of interest, GPR84-like sequences were also found in the sea lamprey *Petromyzon marinus*. Furthermore, cartilaginous fishes such as whale shark (*Rhincodon typus*), Australian ghost shark (*Callorhinchus milii*), and thorny skate (*Amblyraja radiata*) exhibited GPR84-like sequences (30–40% identity to the human or zebrafish GPR84 ortholog, Figure S3). Cartilaginous fishes (sharks, rays, skates) diverged from bony vertebrates about 450 Myr ago, whereas the sea lamprey diverged from the vertebrate lineage approximately 550 Myr ago (Venkatesh et al., 2014; Irisarri et al., 2017; Smith et al., 2018). This confirms the presence of GPR84 in vertebrate genomes since more than 400 Myr and shows that the origin of GPR84 can be dated back to about 550 Myr ago because no GPR84-like sequence could be detected in the genome of lancelets (Figure 1A).

### GPR84 is absent in birds and a pseudogene in bats

Analyses of all vertebrate classes revealed the absence of GPR84 in the class of birds (*Aves*, genomes of at least 61 species available (OBrien et al., 2014; Eöry et al., 2015)) and functional pseudogenization in bats (*Chiroptera*). The GPR84 sequences of 11 different bat species (Figures 1B and S2, Table S1) meet the defining criteria for a pseudogene by showing nonsense and frame-shifting mutations, as well as significantly more non-synonymous substitutions than GPR84 of other species. These non-synonymous substitutions occur also in otherwise very conserved receptor regions (Figure 1B). Ten of 11 bat orthologs showed mutations at loci conserved amongst all other 101 mammalian GPR84 orthologs. Four bat orthologs revealed deletions of at least three amino acid residues in a row, and five bat orthologs exhibited an in-frame stop codon (Figure 1B). These observed features of bat GPR84 orthologs as compared to 101 other mammalian orthologs indicate a loss of selective constraint. To confirm this, we used the RELAX hypothesis testing framework from https://www.datamonkey.org/. RELAX compares a set of test branches (here, the branch of all *Chiroptera*, as shown in Figure S2) with a set of reference branches (here, all other 101 orthologs) and returns a selection intensity parameter k that indicates changes in evolutionary constraint on one subset relative to the other. A k-value significantly greater than 1 indicates intensified selection strength, whereas a significantly lower k-value indicates relaxation. Our analyses revealed a k-value of 0.07 for the *Chiroptera* branch, which was significantly lower than 1 (p< 0.001), indicating a relaxed strength of selection. Owing to the determined loss of constraints on GPR84 in bats, they were not included in our analyses of GPR84 sequence conservation.

### Sequence conservation and specificities in mammalian GPR84

**Figure 2:**
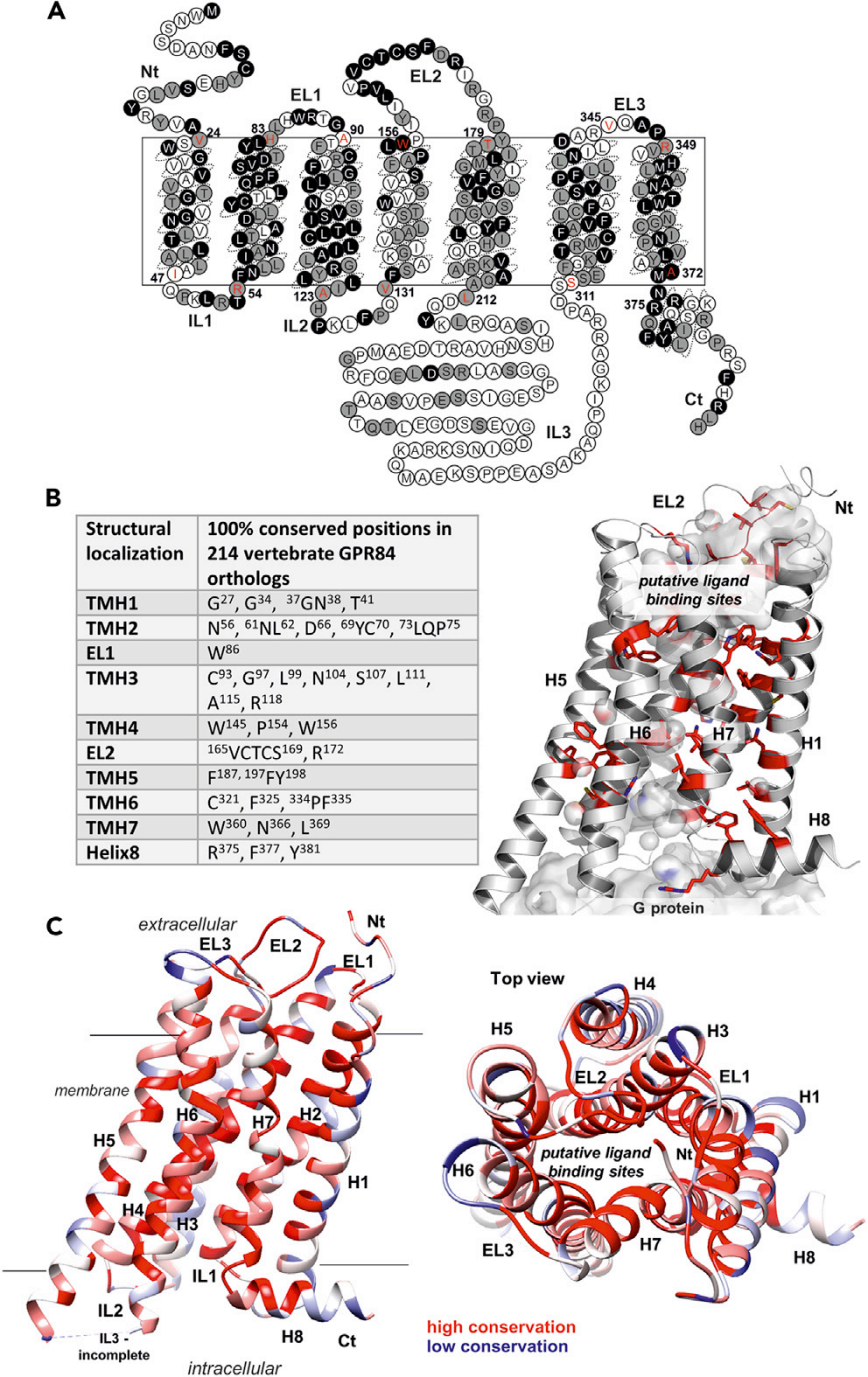
Sequence conservation of GPR84 orthologs (A) Human GPR84 snake-plot highlighting positional conservation among 101 mammalian GPR84 orthologs (see also Table S1). Positions that are 100% conserved are shown in black, positions at which only two or three different amino acids are tolerated are shown in grey, and highly variable positions are shown in white. Indicated in red are the predicted transmembrane helix border positions. (B) Amino acids that are 100% conserved in 214 vertebrate orthologs are listed and depicted in a structural GPR84 model (right) to visualize their spatial distribution. TMH2 and TMH3 are hot spots of conservation and sidechains are mostly directed into the helical core at certain spatial levels (see also Figure S4, Table S2). (C) Conservation of 101 mammalian species visualized at a GPR84 three-dimensional homology model (left - side view, right - top view). The software *Chimera* (Pettersen et al., 2021) was used. Red indicates high conservation of amino acid positions, blue low conservation. The inner core of the transmembrane helical bundle including cover-like arranged parts of the EL2 is most conserved, while membrane-orientated sidechains are less conserved. TMH3 shows the highest conservation in the amino acid composition. IL: intracellular loop, EL: extracellular loop, Ct: *C* terminus, Nt: *N* terminus, H: Helix.

Determination of conserved amino acid positions within a protein can give indications about parts that are of importance for its structure and/or function. For 101 intact mammalian GPR84 orthologs (excluding all bats) sequence conservation was analyzed and the distribution of sequence variations was visualized in a snake plot of human GPR84 (Figure 2A). In general, the conservation of an amino acid residue at a position amongst all orthologs indicates a high evolutionary constraint because no variation at this position has been tolerated. Because this is a spatial as well as structural conservation, both the position and the type of amino acid have to be relevant for protein function and/or structure. Variable positions, on the other hand, occur in regions that are less likely essential for proper protein functionality although they may still play a role for adapted protein functionalities (e.g. in terms of ligand binding), reflecting changing evolutionary constraints.

Analysis of mammalian GPR84 ortholog sequences revealed lower evolutionary conservation specifically in the intracellular loop (IL) 3, extracellular loop (EL) 3 as well as in transmembrane helices (TMH) 1, 4 and 5. The central part of EL2, as well as the TMHs 2, 3, 6, and 7, are highly conserved throughout mammalian GPR84 orthologs (Figure 2A). Although the sequence conservation in non-mammalian vertebrates is much lower (Figure S4), there are several positions in GPR84 that are 100% conserved throughout all vertebrates (Figure 2B). The conserved motif ^165^VCTCS^169^ in EL2 is the most notable because loops are commonly characterized by rather low sequence conservation. This suggests that in GPR84, EL2 is in its structure highly relevant for proper receptor function.

**Figure 3:**
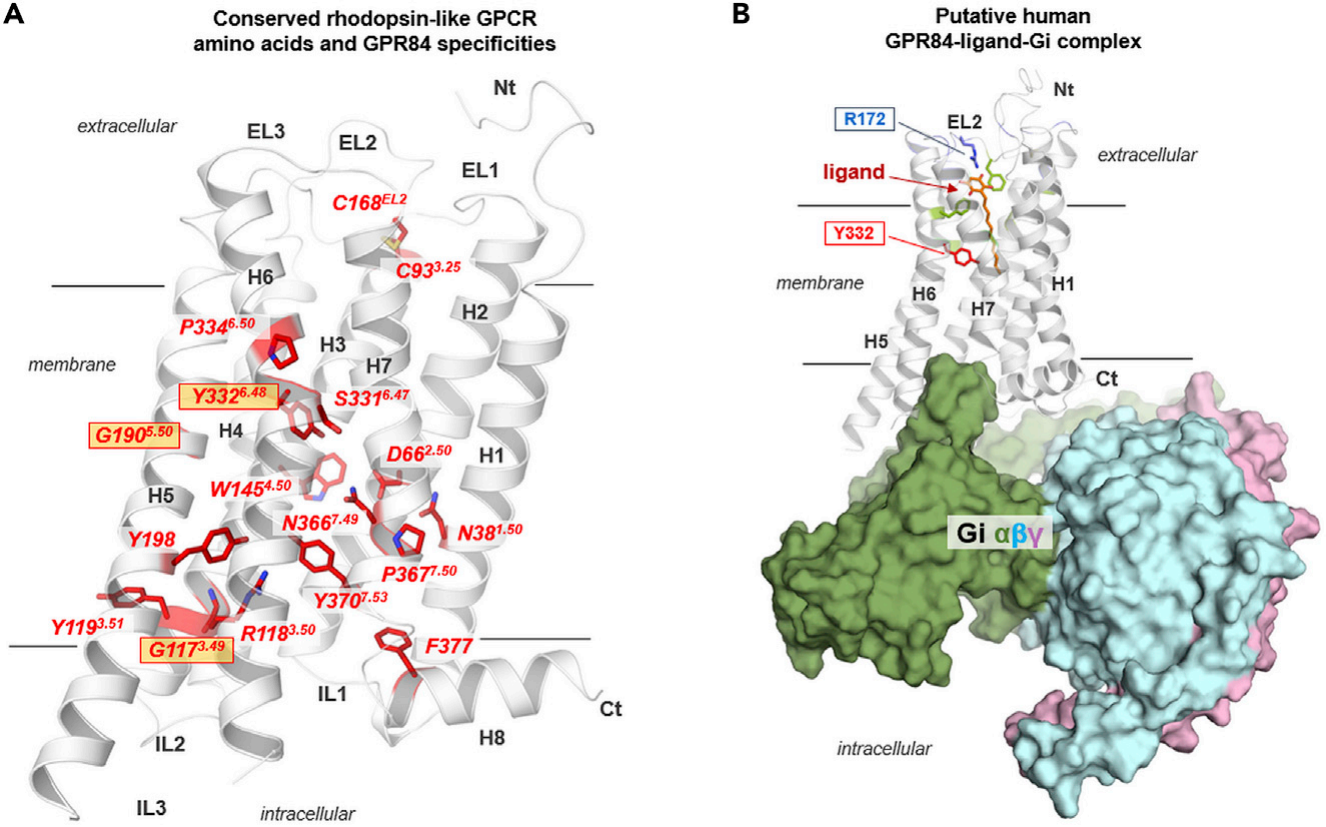
Structural human GPR84 homology model with highlighted positions of conserved rhodopsin-like GPCR positions and an entire GPR84/ligand/G_i_ complex model (A) At the putative inactive receptor state conformation, conserved amino acids are highlighted that are essential for protein fold or the signal transduction process in rhodopsin-like GPCRs (superscript numbers according to Ballesteros & Weinstein numbering (Ballesteros and Weinstein, 1995)). They are often part of motifs, such as the *CPxW* motif in TMH6, or the *DRY* motif in TMH3 (Weis and Kobilka, 2018) (see also Tables S2, S3, S4,and S5). In addition, the disulfide bridge between a cysteine in TMH3 and a cysteine in EL2 stabilizes the EL2 conformation and adjustment above the helical bundle. In GPR84, several usually highly conserved amino acids are different. Examples are the Y332, which is tryptophan in most other rhodopsin-like GPCRs, or the G190, which usually is a proline. Such differences are not unique (Tables S2 and S3), but they are responsible for specificities in the structure and function of GPR84. (B) This model of the fully activated GPR84 complexed with an agonist and the trimeric G protein molecule (G_i_) shows spatial dimensions of interacting molecules and a putative arrangement of the components to each other. This is based on the determined template structures (G_i_ binding) or functional data (ligand binding).

The general conservation pattern mapped on the three-dimensional structural GPR84 model (Figure 2C) revealed that conserved parts cluster together e.g. in helix-helix interfaces or particular amino acid pairings (Figure 2B). The lowest sequence conservation is always observed at the protein-membrane interface (Figure 2C, top view). The inner core of the helical bundle is highly conserved since 550 Myr in all vertebrate GPR84 orthologs because of important functionalities like signal transduction via specific amino acids (Venkatakrishnan et al., 2016), but also because the putative orthosteric ligand-binding site is located here (Figures 2, 3, and S4). Moreover, structural conservation likely indicates the relevance of these positions for correct receptor folding.

GPR84 belongs to the family of rhodopsin-like GPCRs, which consists of more than 650 members (Fredriksson et al., 2003). Within each helix, amino acid positions and motifs have been defined as highly conserved throughout rhodopsin-like GPCRs (Ballesteros and Weinstein, 1995) and have previously been described in detail (Schwartz et al., 2006; Flock et al., 2015). Because GPR84 orthologs exhibit remarkable differences at more than three of such conserved positions or motifs, we analyzed ∼300 rhodopsin-like GPCRs regarding their actual conservation at these positions. Thereby, we interpolated the frequencies of deviation occurring at such specific conserved positions or motifs (Tables S2 and S3).

One of the highly conserved and common motifs for rhodopsin-like GPCRs is the *D(E)RY* motif, which is a *GRY*, *ARY* or *SRY* motif in vertebrate GPR84 orthologs. Our analyses of rhodopsin-like GPCRs showed that the conservation of the aspartate is 64% and of the entire *D(E)RY* motif only about 70% (Tables S2 and S3). Thus, although conserved, four (G: FFAR1, A: GPR62, RXFP4, S: GPR82) out of 286 human rhodopsin-like GPCRs have an uncharged glycine, alanine or serine residue at position 3.49, as has been found in vertebrate GPR84 orthologs (Tables S4 and S5). Similarly, the conservation of the entire *CWxP* motif in TMH6 is 73% and of the *NPxxY* motif in TMH7/Helix H8 58% (Tables S2, S3, S4, and S5), suggesting that divergences in the conserved motifs among rhodopsin-like GPCRs are more common than previously anticipated.

### Functional characterization of mammalian GPR84 orthologs reveals variations in basal activity and ligand responses

**Figure 4:**
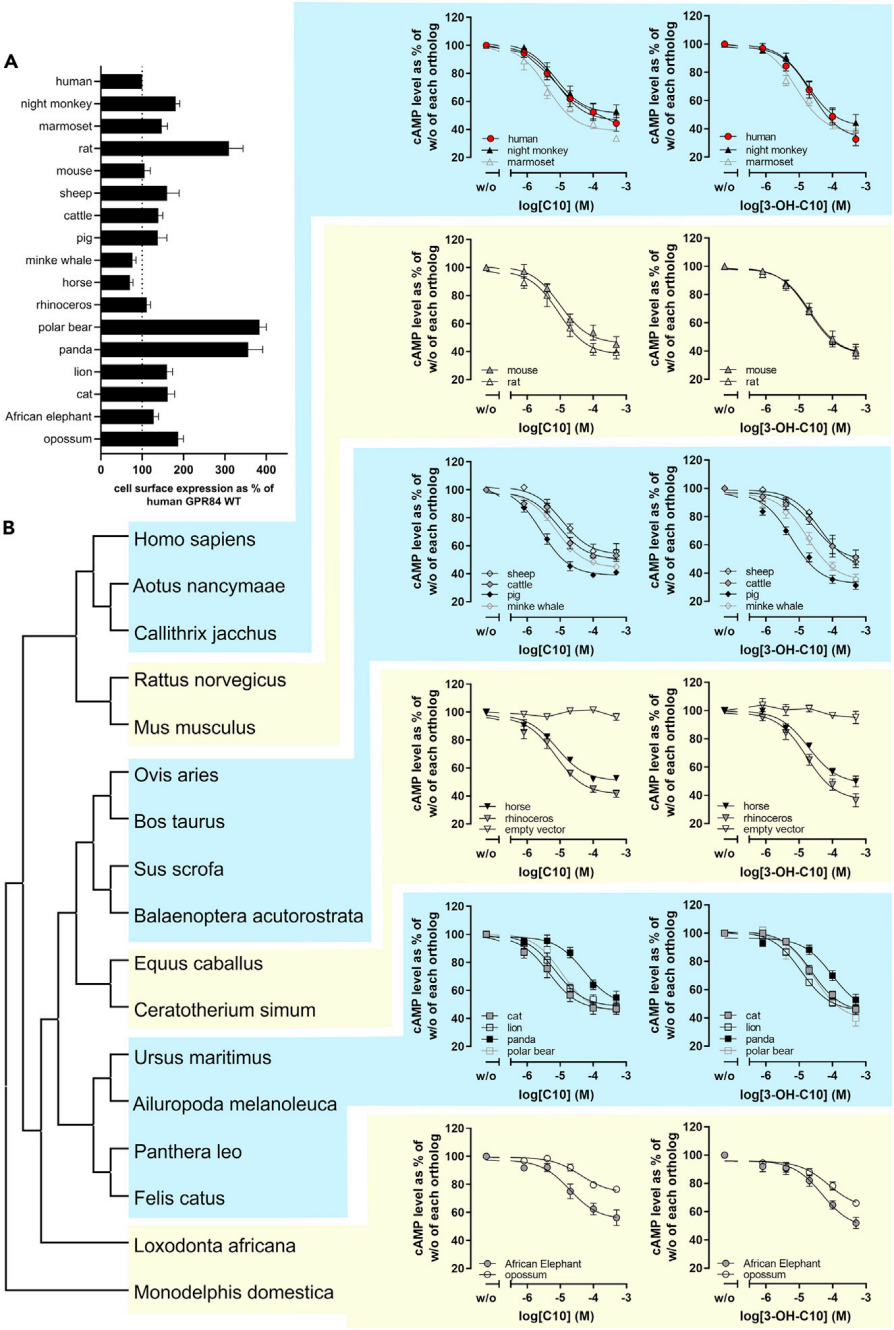
Functional characterization of mammalian GPR84 orthologs CHO-K1 cells were transiently transfected with receptor constructs. (A) Cell surface expression levels of mammalian GPR84 orthologs were measured by ELISA. Specific optical density (OD) readings are given as a percentage of the human GPR84. Data are given as mean ± SEM of at least three independent experiments (see also Table S6). (B) cAMP inhibition assays in presence of 2 μM forskolin were performed showing concentration-dependent activation of mammalian GPR84 orthologs by decanoic acid (C10) and 3-hydroxydecanoic acid (3-OH-C10). cAMP level of each GPR84 ortholog in absence of an agonist was set 100% (E_max_ and EC_50_ values are summarized in Table S7). Data is shown according to the evolutionary relations of the mammals analyzed. See also Figures S5, S6, S7, Tables S8 and S9. Data are given as mean ± SEM of at least three independent experiments.

Besides the structural features and conservation of GPR84 orthologs, we analyzed functional GPR84 properties of selected mammalian species in a heterologous overexpression system. Although CHO-K1 cells lack expression under the natural promoter, immune cell-specific proteins, and species-specific codon usage, such heterologous expression systems provide the only feasible approximation for ortholog comparison. Furthermore, they are widely used in the GPCR field to compare functional receptor properties such as basal activity, ligand affinities, potencies and efficacies. Seventeen of the 101 GPR84 orthologs, representing most of the mammalian orders, including *Primates* (*Platyrrhini* and *Catarrhini*), *Rodentia*, *Carnivora* (*Caniformia* and *Feliformia*), *Cetartiodactyla* (*Cetacea*, *Ruminantia* and *Suina*), *Perissodactyla*, *Afrotheria*, and *Metatheria* (Figure S7), were selected for analyses and comparison of functional properties. These selected species have remarkable differences in their respective habitats, physiology, diets and metabolism (e.g. digestive system). Altogether this results in different microbial challenges for their immune system. We expressed and functionally analyzed these GPR84 orthologs in CHO-K1 cells to gain information about potential species-specific differences in GPR84 signaling capacities in response to C10 and 3-OH-C10. ELISA analyses were carried out to determine whether the mammalian GPR84 orthologs are functionally expressed in CHO-K1 cells, i.e. properly folded and present at the plasma membrane. The determination of transient total expression levels of GPR84 orthologs in CHO-K1 cells revealed that opossum, minke whale and sheep GPR84 showed a lower total expression level compared to the human receptor construct. Significantly higher total expression levels were found for rat, cat, panda and horse GPR84 compared to human GPR84 (Table S6). Rat, polar bear and panda GPR84 exhibited 3- to 4-fold higher cell surface expression as compared to the human GPR84 (Figure 4A).

Previous studies have shown that the human GPR84 exhibits basal signaling activity (Peters et al., 2020). Since GPR84 is known to couple to the Gα_i_ protein, it was tested here, whether the mammalian GPR84 orthologs exhibit constitutive receptor activation, i.e. cAMP inhibition in the absence of ligand. Our data revealed that the 17 mammalian GPR84 orthologs are indeed basally active (Table S6), whereas polar bear, panda, minke whale, and opossum GPR84 showed a significantly lower basal activity than the human ortholog (Table S6).

Compared to the human GPR84, the agonists C10 and 3-OH-C10 activated the GPR84 of opossum and African elephant with significantly higher EC_50_ values, and a lower decrease in cAMP levels (E_max_) was induced (Figure 4B and Table S7). The panda GPR84 exhibited lower potency and efficacy upon stimulation with both agonists, respectively (Figure 4B and Table S7). Among the *Carnivora*, polar bear and cat GPR84 exhibited higher EC_50_ values for 3-OH-C10, but not for C10. The only ortholog activated by C10 and 3-OH-C10 with a lower EC_50_ value was the pig GPR84. Furthermore, 3-OH-C10, but not C10 activated sheep and cattle GPR84, also belonging to the *Cetartiodactyla*, with lower potency (Figure 4B and Table S7).

### Selection analyses of panda and polar bear GPR84 within the *Carnivora* reveal positive selection

Functional differences in basal activity as well as potency were detected within the *Carnivora* for bear GPR84 orthologs (Table S7). Bears evolved 20-25 Myr ago with the giant panda diverging 12-20 Myr ago (Mclellan and Reiner, 1994; Huber and van Manen, 2019). Most bears are omnivorous with polar bear and panda being exceptions representing the extremes of diets within the *Ursidae* (Huber and van Manen, 2019). Polar bears are obligate carnivorous whereas pandas are specialized herbivores (99% of their diet consists of bamboo) (Huber and van Manen, 2019). Here, we tested whether the observed differences in GPR84 function between bears and the *Feliformia* GPR84 orthologs are caused by altered evolutionary constraints. We used aBSREL (adaptive Branch-Site Random Effects Likelihood) to test if positive selection occurred on a proportion of branches (Smith et al., 2015). Among 35 *Carnivora* included in the analyses (Figure S5), aBSREL found evidence of episodic diversifying selection on the node shared by all bears (*Ursidae*) (p = 0.0179, Table S8, Figure S5, blue). On the other hand, the test for selection relaxation using RELAX yielded k = 0.72, which was not significant (p = 0.230, LR = 1.44) for this branch (Wertheim et al., 2015). Further, aBSREL found evidence of episodic diversifying selection on the node shared by bears excluding panda (p = 0.0013, Table S8, Figure S5, green). For this node, RELAX analyses revealed significant selection intensification (k = 2.32, p = 0.012, LR = 6.25) (Figure S5, green) (Wertheim et al., 2015).

Fixed effects likelihood (FEL) analyses, which infer non-synonymous (dN) and synonymous (dS) site-specific substitution rates, were conducted to test for positively selected sites in panda and other bear GPR84 orthologs including polar bear in comparison to all other *Carnivora* (Kosakovsky Pond and Frost, 2005). Evidence was found for pervasive positive/diversifying selection at 18 sites (p value threshold of 0.1 (Table S9, Figure S5, blue). Eight of these 18 sites exhibit bear-specific amino acids that are not present in any other mammalian GPR84 ortholog (Table S9, Figure S6). FEL also identified six positively selected sites present in panda bear, two of which are panda-specific and not found in any other mammalian GPR84, namely Leu^152^ and Ala^249^ (numbering based on panda GPR84). Moreover, on the node shared by *Ursidae* excluding panda, FEL identified seven positively selected sites, including Leu^152^, Lys^171^, Glu^175^ and Val^259^ (Table S9). Manual inspection of all mammalian GPR84 orthologs revealed that at position 175, which is Gly in human GPR84, Asp is found in panda, and as FEL detected, Glu in all other bear GPR84 orthologs. *Ursidae* GPR84 orthologs are the only of the 101 mammalian orthologs that have an amino acid substitution at position 175 (Table S9). In summary, these analyses support that bear GPR84 orthologs are under positive selection.

### Bacterial quorum sensing molecules *cis*-2-decenoic acid (*cis*-2-C10) and *trans*-2-decenoic acid (*trans*-2-C10) activate mammalian GPR84 orthologs with minor differences in potency

**Figure 5:**
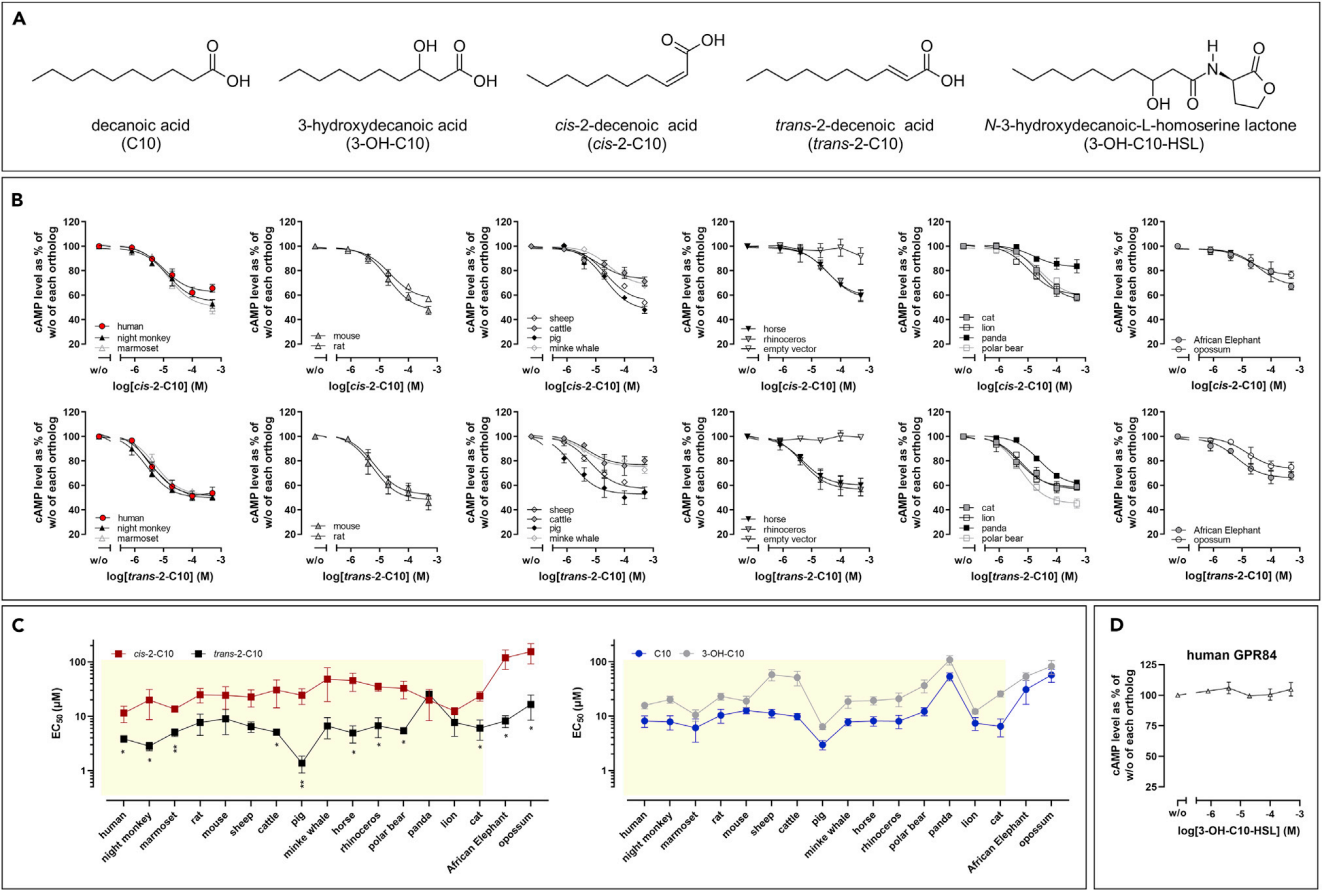
Mammalian GPR84 orthologs are activated by the quorum-sensing molecules *cis*-2-decenoic acid (*cis*-2-C10) and *trans*-2-decenoic acid (*trans*-2-C10) (A) Structures of C10, 3-OH-C10, *cis*-2-C10, *trans*-2-decenoic acid and 3-OH-C10-HSL. (B) CHO-K1 cells were transiently transfected with receptor constructs. GPR84 orthologs were stimulated with *cis*-2-C10 or *trans*-2-C10 (E_max_ and EC_50_ values are summarized in Table S7). (C) EC_50_ values of *cis*-2-C10, *trans*-2-C10, C10 and 3-OH-C10 are visualized. Unpaired two-tailed t-tests were used to compare EC_50_ values of *cis*-2-C10 to EC_50_ values of *trans*-2-C10 within each GPR84 ortholog (see also Table S7). Highlighted in yellow are *Boreoeutheria* species for which the least variance in EC_50_ values for *cis*-2-C10 is observed. The African elephant belongs to *Afrotheria* and opossum to *Metatheria*. (D) 3-OH-C10-HSL does not induce a concentration-depended response in cAMP inhibition assays of human GPR84. See also Figure S7. (B, C, D) Data are shown as mean ± SEM of at least three independent experiments.

GPR84 officially remains an orphan receptor because of the low concentration of MCFAs and 3 OH-MCFAs *in vivo*. Thus, we reasoned that the observed changes of selective pressure on mammalian GPR84 orthologs may be caused by formerly unrecognized biological sources (plants, bacteria, and fungi) of GPR84 ligands. Previous studies have shown that 3-OH-MCFAs, including 3-OH-C10, are components of LPS present in the outer membrane of gram-negative bacteria and may serve as endotoxin markers in clinical samples (Szponar et al., 2002, 2003; Park et al., 2004; DeLa Cochetiere et al., 2009). Considering GPR84 as a pro-inflammatory immune cell receptor, we hypothesized that other bacteria-derived metabolites may act as agonists of GPR84. Several structurally to 3-OH-MCFAs-related bacterial compounds are known, all of which are essential for quorum sensing (QS), i.e., communication in bacteria. QS molecules are produced and released by bacteria as chemical signal molecules in response to fluctuations in cell-population density (Miller and Bassler, 2001; Papenfort and Bassler, 2016). Here, we tested, whether the QS molecules *cis*-2-C10, *trans*-2-C10 and *N*-3-hydroxydecanoic-L-homoserine lactone (3-OH-C10-HSL) act as ligands activating GPR84 (Figure 5A).

Using cAMP inhibition assays, we demonstrated that all mammalian GPR84 orthologs are activated by *cis*-2-C10 and *trans*-2-C10 (Figure 5B and Table S7). Human GPR84 is activated by *cis*-2-C10 with a potency of ∼12 μM and by *trans*-2-C10 with an EC_50_ of ∼4 μM. At most but not all GPR84 orthologs the potency of the *trans*-isomer is significantly higher than that of the *cis*-isomer (Figure 5B, Figure 5C and Table S7). Moreover, a comparison of EC_50_ values of C10, 3-OH-C10, *cis*-2-C10 and *trans*-2-C10 across mammalian orthologs revealed that *cis*-2-C10 is the least variable GPR84 agonist at *boroeutherian* GPR84 orthologs (Table S7, Figure 5C). EC_50_ values of *cis*-2-C10 varied only by factor four across *boroeutherian* GPR84 orthologs as compared to factors 18, 17 and 19 for C10, 3-OH-C10 and *trans*-2-C10, respectively (Table S7, Figure 5C). 3-OH-C10-HSL did not activate the human GPR84 (Figure 5D).

### Frequency analyses and functional characterization of naturally in human GPR84 occurring variants

Evolutionary conservation analyses of GPR84 revealed unique adaptations in different mammalian orders. Variations in GPR84 also occur within human populations. Thus, we analyzed the occurrence, frequency, and functional consequence of naturally occurring GPR84 variants. We hypothesized that these analyses will reveal the impact of SNPs occurring in human GPR84 on its function and potentially indicate populations with GPR84 underlying changing evolutionary constraints.

The dbSNP database (Sherry et al., 2001) and the *Catalogue of Somatic Mutations in Cancer* (COSMIC) (Tate et al., 2019) revealed 275 SNPs in human GPR84 causing an altered coding sequence. No direct relation of GPR84 variants with any disease or pathogenic condition is evidenced so far. Here, we analyzed 33 of these naturally occurring GPR84 variants, five of which represent the most frequent heterozygously occurring SNPs in GPR84 according to the dbSNP database (Table S10).

Because three of these SNPs (rs77767409: S15Y, rs11170883 G37D, rs77759698: Y370H) occurred with a minor allele frequency (MAF) > 1% among East Asian populations, we analyzed the GenomeAsia 100K database (GenomeAsia100K Consortium, 2019) to get further insights in the distribution of these SNPs in GPR84. The Indonesian population groups exhibited a discernibly higher MAF for Y370H: the Mentawai (MEN, 20%), the Nias (NIA, 10%) and Bena Flores (BEN, 4.5%) (Table S10). NIA also revealed a high MAF (10%) for S15Y. The Northeast Asian populations, such as the Han from China (HAN), Japanese (JPN), and Koreans (KOR) showed a high MAF for G37D (HAN: 13.6%, JPN, 3.3%, KOR: 4.3%) and Y370H (HAN: 4.5%, JPN: 3.3%, KOR: 3%) (Table S10). Identity by descent (IBD) analyses were performed to assess selection within populations. Alleles are considered to be IBD if they are inherited from the same ancestral allele. Therefore, the loci with high IBD sharing can be used as the proxy for high selection pressure on them, compared to what can be expected under neutrality (Albrechtsen et al., 2010). All 15 NIA samples from Indonesian islands were found to share the highest IBD (pi-Hat = 1) for the three SNPs (rs77767409: S15Y, rs11170883 G37D, rs77759698: Y370H) that have MAF >1% among East Asians. This indicates strong selection pressure on these variants potentially to preserve the non-functional minor alleles.

**Figure 6:**
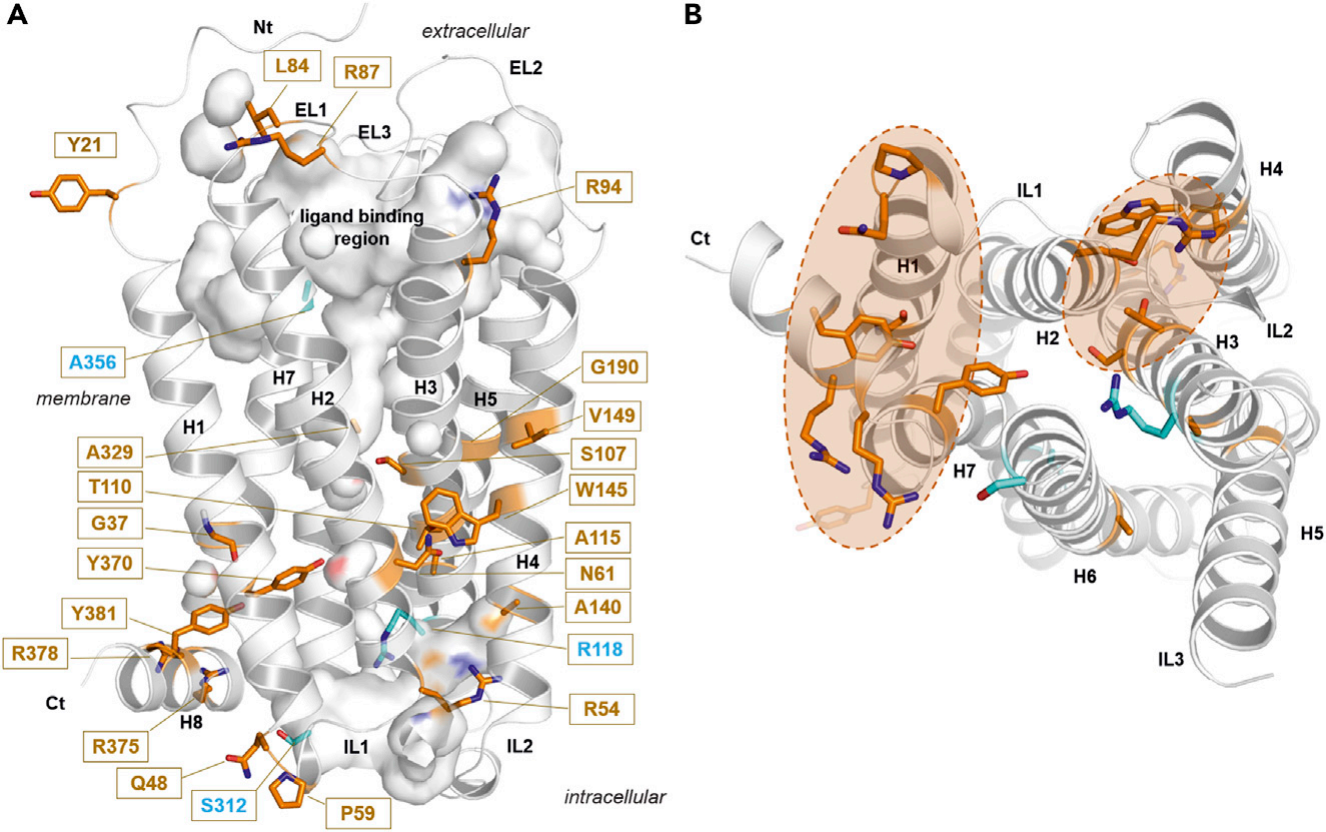
Positions of naturally occurring variants (SNPs) mapped on a structural GPR84 model (A) The homology model of GPR84 in an inactive state conformation revealed insight into the spatial distribution of human GPR84 variants investigated in this study. Respective functional impact on receptor functions is indicated by different colors: brown - expression decreased, cyan - signaling decreased (EC_50_) (Table S10). This visualization combines sequence information, functionalities, and structural information, leading to the conclusion, that human GPR84 variations are clustered spatially in two main regions. (B) View from the cytosolic side: Besides three exceptions (R118 in TMH3, S312 in TMH6 likely involved in G protein-binding, and A336 located close to the binding site), two hotspots of amino acid variations causing a decrease in cell surface expression of GPR84 were identified. They are located (1) at the intracellular interface between helix H8, TMH1 and TMH7, and (2) at the shared interface between TMHs 2-3-4 (brown translucent circles).

The remaining 28 variants are somatic missense mutations reported to occur heterozygously in tissues of carcinoma of breast, kidney, lung, liver and large intestine. Cell surface expression levels and response to C10 were determined in cAMP inhibitions assays of 33 GPR84 missense variants distributed across the receptor (Table S10). These analyses revealed wild-type-like human GPR84 function for nine variants. Most of these variants are located in the IL3, one in TMH7, and one in the *C* terminus (Figure 6, Table S10). A reduced plasma cell membrane expression was found for 22 of the studied GPR84 variants indicating potential receptor inactivation, although nine of them still responded to C10 (Table S10). The R118C and R118H, both affecting the arginine of the *GRY* motif in TMH3, exhibited no disturbed cell surface expression but did not respond to C10 (Figure 3, Table S10). This is in accordance with the known importance of this highly conserved arginine (Arg 3.50) for receptor functionality and G protein-coupling in rhodopsin-like GPCRs (Scheerer et al., 2008).

In the three-dimensional GPR84 model (Figure 6), besides a few exceptions on the extracellular side (e.g. Y21H, R87C, R94M), there is a clear separation of two hotspot regions leading to a decrease in cell surface expression of GPR84: (1) at the intracellular interface between helix H8, TMH1 and TMH7, and (2) at the shared transmembrane interface between TMHs 2-3-4. These amino acid residues are important for correct receptor folding and receptor internalization or trafficking to the cell surface (e.g., A140V, W145C). Some of these GPR84 variants are still activated by C10 because they do not affect the ligand-binding region (Figure 6). A356V is located close to the ligand-binding site. However, we conclude that the identified GPR84 variants are unlikely to have wild-type function *in vivo*.

## Discussion

GPCRs and accompanying effector proteins like G proteins arose ∼1000 Myr ago (Römpler et al., 2007). GPR84 is an evolutionary old receptor already present in early vertebrates (∼550 Myr ago). Here, we studied GPR84 in vertebrates with a focus on mammals to gain information about putatively changing evolutionary constraints, crucial structural regions, and potential differences in receptor function. The acquired data, in combination with publicly available knowledge about habitat, diet, diet-associated bacterial load, the digestive and immune system of the analyzed mammalian species may enable an improved understanding of the physiological role of GPR84.

### Mammalian GPR84 orthologs functionally differ, with GPR84 of bears showing signatures of positive selection

The analyses of functional properties of mammalian GPR84 orthologs in a heterologous expression system revealed several deviations in the parameters cell surface receptor expression, basal activity, and ligand-induced signaling, most strikingly for panda and polar bear GPR84 (Tables S6 and S7). Both exhibited decreased basal activity, whereas in parallel significantly more receptor was detectable at the plasma membrane. This is likely because of a decreased internalization rate in the absence of ligand caused by specific amino acid residues in the *Ursidae* GPR84 orthologs (Table S9, Figure S6). A cluster of five bear-specific residues is located at the transitions of the EL2 to adjacent helices probably causing the down-regulated basal activity. For many GPCRs, the EL2 is known to be essential for the regulation of conformations associated with different activity states (Massotte and Kieffer, 2005; Woolley and Conner, 2017), including basal activity propagation (Kleinau et al., 2007). This leads to the question: Which GPR84-associated physiological (immunological) functions are potentially related to the observed constitutive activity of mammalian GPR84 orthologs?

Many GPCRs have been reported to exhibit basal activity in heterologous overexpression systems (Seifert and Wenzel-Seifert, 2002; Bond and Ijzerman, 2006). Basal activity affects functionalities such as ligand affinity (lowering of the energetic barrier for activation), or mediates a persistent tonus of signaling (lowering the “energetic costs” of constitutive physiological processes) (Kleinau and Biebermann, 2014). Furthermore, constitutive receptor activity is dependent on the ease of receptor isomerization from an inactive to an active conformation, and the efficiency of receptor-effector coupling in the cell (Berg and Clarke, 2018). In the case of GPR84, a receptor expressed in innate immune cells forming the first-line defense against invading microorganisms, the constitutive activity might serve as a guardian protecting against invading microorganisms. The constitutive activity of the Gα_i_-coupled CC-type chemokine receptor 1 (CCR1) has been shown to induce cellular responses like basal migration of leukocytes (Gilliland et al., 2013). Furthermore, CCR1-β-arrestin-2 complexes are relevant for receptor responsiveness and maintenance of chemokine gradients during inflammation (Gilliland et al., 2013). Similarly, GPR84 constitutive activity could allow for directional detection of microbial-derived metabolites. The immune response is a tightly controlled process, sufficient to protect from pathogens but limited enough to protect from excessive damage of surrounding tissue. Thus, GPR84 basal activity may be linked to processes like neutrophil swarming or its self-limitation (Kienle et al., 2021). At last, naturally occurring inverse agonists of endogenous or exogenous origin reducing the constitutive receptor activity of GPR84 might exist *in vivo* but have yet to be discovered. In summary, basal activity of human-encoded immune cell GPCRs, like GPR84, serve presumably diverse functions.

We conclude from our analyses that the reduced basal activity of bear GPR84 orthologs is likely a trait acquired in the last common ancestor of all *Ursidae* (Figure S5, Tables S8 and S9). *Ursidae* are geographically widespread and occupy a broad range of ecologic niches (Collins, 2015; Kumar et al., 2017). Thus, it is difficult to attribute a trait shared by all bears to be associated with the lower basal activity observed for GPR84. A physiological process of bears in the northern hemisphere that has been shown to suppress the innate immune system is torpor (Sahdo et al., 2013), but it is unknown whether the last common ancestor of bears was entering torpor. One hypothesis is that a colder climate inhabited by the last common ancestor of *Ursidae* was associated with less infectious agents, which caused adaptations of the innate immune response also affecting the basal activity of GPR84.

Panda GPR84 showed significantly lower potencies for the ligands C10, 3-OH-C10 and *trans*-2-C10 than human GPR84. The bear-specific amino acid cluster at the EL2/TMH5 transition (Figure S6) and the panda bear GPR84-specific Asp^175^ (Glu in other *Ursidae*) likely have an impact on the justification of Arg^172^ towards the ligand-binding site (Figure 3B) thus affecting ligand potencies. The Arg^172^ (EL2) in GPR84 has previously been shown to be a direct interaction partner for certain ligands (Jenkins et al., 2021). Furthermore, in panda GPR84 the interface between TMH3 and TMH4 is modified by two interacting panda-specific amino acids (Thr^102^, Leu^152^) at the same spatial layer (Figure S6). Such positions affect protein functionality because the adjustment of helical interfaces is crucial for receptor properties (Magnani et al., 2016; Bibbe and Vriend, 2021). This may account for the low potency of ligands at panda GPR84. Our findings suggest that positive selection shaped panda GPR84 evolution although the responsible selective constraints remain elusive (Tables S6, S7, S8, and S9, Figure 4). However, compared to other *Ursidae*, the panda is strictly herbivorous (Huber and van Manen, 2019) thus experiencing a much lower exposition to pathogens that infect mammals as compared to other bears.

One other notable ortholog is pig GPR84, which exhibited a significantly higher potency for C10, 3-OH-C10, and *trans*-2-C10 than human GPR84 (Table S7, Figure 4). Comparing these potencies to the other GPR84 orthologs tested from the mammalian order *Cetartiodactyla*, it becomes apparent that one specialty of the pig is its omnivorous diet as opposed to an exclusively herbivorous diet in sheep and cattle. However, efficacies of all tested ligands are variable in *Cetartiodactyla* GPR84 orthologs, suggesting different evolutionary adaptations to unknown selective constraints (Table S7, Figure 4).

### Naturally occurring human GPR84 variants

Evolutionary analyses of GPR84 revealed unique features and adaptations in different mammalian orders. Thus, we also analyzed the occurrence, frequency, and functional consequence of naturally occurring human GPR84 variants. These studies were motivated by the following hypotheses: (1) All naturally in human occurring GPR84 variants at evolutionary highly conserved positions negatively impact GPR84 function. (2) Analyses of the most frequently in human occurring SNPs (Sherry et al., 2001) and their functional relevance may indicate populations in which GPR84 is under a selective constraint allowing for interpolation of associated phenotypes.

For 22 of the studied GPR84 variants, a significantly reduced plasma membrane expression was measured (Table S10), indicating that these 22 amino acid positions are important for correct receptor folding, expression, or trafficking to the cell surface. Most but not all of these variants indeed occur at evolutionarily conserved positions. From a structural perspective (Figure 6), the receptor variants with a decreased cell surface expression level cumulate in two spatial regions: (1) The intracellular interface between TMH1-TMH7-helix H8, and (2) between TMHs 2-3-4 at the intracellular side. Although nine of the 22 GPR84 variants still responded to C10 (Table S10), most of the analyzed GPR84 variants likely do not exhibit wild-type function *in vivo*.

Regarding our second hypothesis, four of the five SNPs occur with the highest frequency in Asia (Table S10). We analyzed the GenomeAsia 100K database (GenomeAsia100K Consortium, 2019) to get further insight into the distribution of SNPs causing amino acid changes in GPR84. Inherited polymorphisms occurring within and among populations can be associated with a genetic trait or a phenotype linked to the presence of an exogenous (environmental) stimulus (Brookes, 1999; Rebbeck et al., 2004; Hirschhorn and Daly, 2005). It is further established that SNPs can influence the immune response to pathogenic challenges and disease outcomes, which in sum contributes to a range of susceptibility to infections among individuals and populations (Martin et al., 2003). Thus, SNPs in human GPR84 may have a protective role, influence disease progression, or even the type of cellular immune response evoked by pathogens, as has been shown for other immune-modulatory genes (Hill, 2001; Skevaki et al., 2015). In several Indonesian population groups, a higher allele frequency of the SNPs Y370H and S15Y was detected suggesting that these non-functional minor alleles are preserved (Table S10). As previously described, the Indonesian populations have been exposed to strong selective pressures exerted on the immune system over thousands of years by diverse infectious disease-causing human pathogens (Natri et al., 2020). Similarly, the Northeast Asian populations of Han Chinese, Japanese and Koreans showed a high frequency of SNPs altering GPR84 function. The frequency of these GPR84 SNPs potentially increased because they were beneficial and improved host survival (Quintana-Murci, 2019).

### Implications of GPR84 sequence specificities in conserved rhodopsin-like GPCR motifs

Mammalian GPR84 orthologs remarkably differ in three amino acid positions compared to most other rhodopsin-like GPCRs (Figure 3A): 1. A glycine at position 3.49 (aspartate or glutamate in 85% rhodopsin-like GPCRs), 2. A glycine at position 5.50 in TMH5 (proline in 79% rhodopsin-like GPCRs) and 3. A tyrosine at position 6.48 in TMH6 (tryptophan in 68% rhodopsin-like GPCRs) (Tables S2 and S3).

The *D(E)RY*-motif, common for rhodopsin-like GPCRs, is a *GRY*-motif (Figures 2A and 3) in most mammalian GPR84 orthologs, whereas all 35 species of the order *Carnivora* exhibit an *ARY* motif (Figure S5). In most non-mammalian vertebrate GPR84 orthologs, an *SRY* motif is present (Table S2). For several GPCRs, negatively charged aspartate (D) or glutamate (E) amino acid residues in this motif are crucial for intramolecular interactions between TMH3 and IL2, both required for G protein activation (Hofmann et al., 2009; Fanelli and Benedetti, 2011). Thus, an uncharged glycine, alanine, or serine residue at position 3.49 in GPR84 likely affects the receptor/G protein interplay.

The Pro 5.50 in TMH5 is related to a structural kink and bulge, which causes a non-regular helix conformation instead of a straight and stable alpha-helix (Yohannan et al., 2004). Such kinks are important in membrane proteins like GPCRs because they create weak points in the helix to facilitate movements that are e.g. associated with receptor activation (Reiersen and Rees, 2001; Yohannan et al., 2004). Consequently, GPR84 is different by lacking such a kink, resulting in a straight TMH5 that has a further impact on several receptor properties, like the exact constitution of the ligand-binding site as shown recently for the melanocortin receptor 4 (Heyder et al., 2021).

Another GPR84 specificity, compared to most rhodopsin-like GPCRs, is a tyrosine instead of tryptophan at position 6.48 in TMH6 in all here analyzed mammalian GPR84 sequences. Some non-mammalian GPR84 vertebrate orthologs have a Phe 6.48 (75 out of 113 species, Table S2). The Trp 6.48 is part of the conserved *CWxP* motif that participates in the stabilization of an active state conformation (Scheerer et al., 2008). In addition or complementation, mammalian GPR84 orthologs exhibit a conserved serine at adjacent position 6.47 instead of the cysteine in this motif, whereas most of the non-mammalian vertebrate GPR84 orthologs have a canonical Cys 6.47 (∼71% conserved in rhodopsin-like GPCRs, serine occurs in ∼10%, Table S2). Thus, the positions 6.47 and 6.48 differ remarkably between mammalian and non-mammalian vertebrate GPR84 orthologs. In conclusion, mammalian GPR84 orthologs possess a very unusual *SYxP* instead of a *CWxP* motif, and most non-mammalian vertebrates have a *CFxP* motif (Table S3). This may affect the basal activity or agonist-induced activation of downstream G proteins by GPR84. The here highlighted peculiarities of GPR84 await deeper experimental analyses to further decipher its molecular properties and specificities.

### Bacterial quorum sensing molecules activate mammalian GPR84 orthologs consistently

In immune cells, GPR84 expression is up-regulated upon stimulation of Toll-like receptor (TLR) 4 by gram-negative bacteria-derived LPS, or upon activation of TLR2 by gram-positive bacteria- and yeast-derived metabolites (Recio et al., 2018). Bacteria can sense and respond to changes in their populations through communication via small signaling molecules, a process called quorum sensing (QS) (Zhou et al., 2017). Representatives of the diffusible signal factor (DSF) family of QS signaling molecules have been found in diverse gram-negative bacterial pathogens (Deng et al., 2011; Ryan et al., 2015). *Cis*-2-C10 (DSF) and *trans*-2-C10 (Streptococcus DSF, SDSF) are known as inter-kingdom signaling molecules originating from gram-negative and gram-positive bacteria, respectively (Vílchez et al., 2010; Marques et al., 2015; Rahmani-Badi et al., 2016; Zhou et al., 2017).

Our functional analyses of mammalian GPR84 orthologs with these bacterial QS molecules revealed them as highly potent ligands (Figure 5C), with no significant differences in potencies of *cis*-2-C10 when comparing *boroeutherian* GPR84 orthologs (Figure 5C). The potencies of *trans*-2-C10 exhibited a similar profile to C10 at most mammalian GPR84 orthologs (Figure 5C). Therefore, our data suggest that GPR84 might have evolved as a receptor with the conserved function to recognize bacterial QS molecules like *cis*-2-C10. GPR84 is a known enhancer of inflammation. Thus, it appears plausible that GPR84-expressing innate immune cells, recruited to the site of infection, induce further pro-inflammatory responses when the bacterial load, and with that the concentration of GPR84-activating QS molecules, is high. If this scenario proves reasonable, GPR84 serves as a sensor for local bacterial load during infection. Unfortunately, local concentrations of *cis*-2-C10 and *trans*-2-C10 at the side of bacterial infections are unknown so far but should be studied in detail together with GPR84-related functionalities *in vivo*.

### GPR84 is non-functional in bats – Does flying make the difference?

GPR84 is already present in cartilaginous fishes (Figures S1 andS3) but absent in the whole class of birds (*Aves*) and a pseudogene in all bats (*Chiroptera*) (Figure 1). Because we were unable to identify a common pseudogenization event in bats, GPR84 inactivation occurred independently after the split of the various bat species (Figure 1B). This leads to the question: Why have bats lost GPR84?

Bats are comprised of a wide variety of species, colonizing diverse habitats, differing in size and dietary requirements (carnivorous, herbivorous, frugivorous, omnivorous), thus physiology. Bats can fly associated with miniaturized body size, an enhanced metabolic rate, and antioxidant capacity (reviewed in (Nabi et al., 2021)). Bats possess unique immunological features and are natural reservoirs for a diverse range of viruses (reviewed in (Nabi et al., 2021)). Over 70 different viruses are carried by bats, most of which do not cause disease symptoms (Lu et al., 2020). This is because of better control of inflammation while combating the virus, hence preventing hyper-inflammation that otherwise exacerbates disease phenotypes and contributes to mortality (Gorbunova et al., 2020).

GPR84 has been linked to increased adhesion of immune cells to bacterial cell walls and phagocytosis (Recio et al., 2018). Assuming that GPR84 evolved as a GPCR supporting anti-bacterial and inflammatory immune responses, a lack of the receptor could be associated with a higher susceptibility to bacterial pathogens. But why should that be advantageous? For any organism, the immune response is a metabolic costly activity. The same is true for the ability to fly. In bats, the increased metabolic activity because of flight is accompanied by the capability to suppress inflammation (Subudhi et al., 2019). Viral infections are often associated with a loss of barrier integrity like the intestinal or the airway epithelium (Chelakkot et al., 2018; Suzuki, 2020; Linfield et al., 2021). This viral disruption of epithelial barrier function could be associated with a higher load of QS molecules activating GPR84, which may cause hyper-inflammation. In humans, many pathogenic RNA viruses have in common that the host immune response contributes to the disease process (Mandl et al., 2015). In this context, one particularly important driver of tissue damage during infection is a dysregulated and excessive innate immune response (Mandl et al., 2018). Thus, loss of GPR84 in bats might be part of the mechanism protecting against excessive inflammation in response to the increased epithelial permeability caused by a viral infection and accompanied by invasion of bacterial pathogens (Chelakkot et al., 2018; Suzuki, 2020; Linfield et al., 2021). The higher potential susceptibility to bacterial pathogens could be circumvented by adaptations of the adaptive immune responses in bats, rendering GPR84 dispensable. At the genomic level, bats have a much larger repertoire of germline genes encoding immunoglobulin segments than humans, which might provide a larger number of antigen-specificities in their naive B cell receptor repertoire (Banerjee et al., 2020). Thus, loss of GPR84 in bats may have been advantageous by protection from excessive inflammation and adaptions of the adaptive immune response to protect from bacterial pathogens.

### Conclusions

In the present study, we systematically addressed structural and functional aspects of GPR84 vertebrate evolution. We investigated a potential link between GPR84 function and habitat or lifestyle across a broad range of mammalian species. While being mostly conserved, we found changing evolutionary constraints in bear GPR84 orthologs reflected in positively selected sites. Moreover, we identified naturally occurring human GPR84 variants causing a loss of function, with increased allele frequency in some Indonesian and some Northeast Asian populations. This suggests that these GPR84 variants arose from beneficial mutations that improved host survival although the stimulus exerting the selective pressure remains elusive.

In summary, our results suggest that infectious disease-causing microbial pathogens exert selective pressures on GPR84 as an immune cell receptor conserved for the recognition of bacteria-derived QS molecules. Finally, the acquired data highlights unique molecular and structural features of GPR84, which opens up questions regarding their role in ligand recognition and signal transduction. Drug discovery efforts may benefit from the improved functional and molecular understanding of GPR84 provided in our study.

### Limitations of the study

Although our study reveals differences in functional properties of mammalian GPR84 orthologs, it does not provide these results within the original species, within immune cells that mainly express GPR84 or under the natural promoter, which would better reflect endogenous expression levels. Future studies may be carried out with primary freshly isolated innate immune cells of different species to get a better understanding of GPR84 function at endogenous expression levels. Furthermore, GPR84 may play a role in the determination of digestive niches, which is likely associated with differences in dietary as well as gut microbiota. However, at present, our data and sample size are not sufficient to draw such conclusions. In addition, more information about concentrations of the bacterial quorum sensing molecules *cis*-2-C10 and *trans*-2-C10 in different diets, the gut, and at sites of infection is urgently needed. At last, although our study highlights the structural peculiarities of GPR84, very detailed experimental analyses are needed to decipher their role for receptor function, which would have exceeded the scope of the present study.

## STAR★Methods

### Key resources table

**Table:** 

REAGENT or RESOURCE	SOURCE	IDENTIFIER
**Antibodies**		

Anti-HA-Peroxidase, High Affinity from rat IgG1	Sigma-Aldrich	12013819001RRID: AB_390917
monoclonal Anti-FLAG M2 antibody produced in mouse	Sigma-Aldrich	F3165RRID: AB_259529

**Bacterial and virus strains**		

NEB 5-alpha competent *E.coli*	NEB	C2987

**Biological samples**		

genomic DNA see Table S11 for species	see Table S11 for sources	N/A

**Chemicals, peptides, and recombinant proteins**		

Q5 High-Fidelity DNA Polymerase	NEB	M0491
Phusion High-Fidelity DNA Polymerase	Thermo Fisher Scientific	F-530
Lipofectamine 2000	Thermo Fisher Scientific	11668019
Penicillin-Streptomycin	Thermo Fisher Scientific	15140122
Fetal Bovine Serum	Thermo Fisher Scientific	10270106
3-isobutyl-1-methylxanthine	Sigma Aldrich	I5879
forskolin	Sigma Aldrich	F3917
decanoic acid	Sigma Aldrich	C1875
(±)-3-hydroxydecanoic acid	Sigma Aldrich	H3648
cis-2-decenoic Acid	Cayman Chemical	Cay11966
trans-2-decenoic Acid	Cayman Chemical	Cay17888
N-3-hydroxydecanoyl-L-homoserine lactone	Cayman Chemical	Cay9001147

**Critical commercial assays**		

AlphaScreen cAMP assay kit	PerkinElmer Life Sciences	6760635M

**Deposited data**		

mammalian GPR84 ortholog sequences generated in this paper, Table S1	NCBI	see Table S1
vertebrate GPR84 ortholog sequences mined from NCBI database	NCBI	see Table S1

**Experimental models: Cell lines**		

CHO-K1 cells	ATCC	CCL-61 RRID: CVCL_0214

**Oligonucleotides**		

see Table S12 for sequences	SeqLab	N/A

**Recombinant DNA**		

HA-/FLAG-tagged Human GPR84 in pcDps	(Peters et al., 2020)	N/A
HA-/FLAG-tagged Azara‘s night monkey GPR84 in pcDps	This paper	N/A
HA-/FLAG-tagged Common marmoset in pcDps	This paper	N/A
HA-/FLAG-tagged Brown rat in pcDps	This paper	N/A
HA-/FLAG-tagged House mouse in pcDps	This paper	N/A
HA-/FLAG-tagged Sheep in pcDps	This paper	N/A
HA-/FLAG-tagged Cattle in pcDps	This paper	N/A
HA-/FLAG-tagged Pig in pcDps	This paper	N/A
HA-/FLAG-tagged Minke whale in pcDps	This paper	N/A
HA-/FLAG-tagged Horse in pcDps	This paper	N/A
HA-/FLAG-tagged White rhinoceros in pcDps s	This paper	N/A
HA-/FLAG-tagged Polar bear in pcDps	This paper	N/A
HA-/FLAG-tagged Giant panda in pcDps	This paper	N/A
HA-/FLAG-tagged Lion in pcDps	This paper	N/A
HA-/FLAG-tagged Cat in pcDps	This paper	N/A
HA-/FLAG-tagged African elephant in pcDps	This paper	N/A
HA-/FLAG-tagged Gray short-tailed opossum in pcDps	This paper	N/A
HA-/FLAG-tagged Human GPR84 S15Y in pcDps	This paper	N/A
HA-/FLAG-tagged Human GPR84 Y21H in pcDps	This paper	N/A
HA-/FLAG-tagged Human GPR84 G37D in pcDps	This paper	N/A
HA-/FLAG-tagged Human GPR84 Q48K in pcDps	This paper	N/A
HA-/FLAG-tagged Human GPR84 P49S in pcDps	This paper	N/A
HA-/FLAG-tagged Human GPR84 R54Q in pcDps	This paper	N/A
HA-/FLAG-tagged Human GPR84 N61S in pcDps	This paper	N/A
HA-/FLAG-tagged Human GPR84 L84M in pcDps	This paper	N/A
HA-/FLAG-tagged Human GPR84 R87C in pcDps	This paper	N/A
HA-/FLAG-tagged Human GPR84 R94M in pcDps	This paper	N/A
HA-/FLAG-tagged Human GPR84 S107P in pcDps	This paper	N/A
HA-/FLAG-tagged Human GPR84 T110N in pcDps	This paper	N/A
HA-/FLAG-tagged Human GPR84 A115T in pcDps	This paper	N/A
HA-/FLAG-tagged Human GPR84 R118C in pcDps	This paper	N/A
HA-/FLAG-tagged Human GPR84 R118H in pcDps	This paper	N/A
HA-/FLAG-tagged Human GPR84 A140V in pcDps	This paper	N/A
HA-/FLAG-tagged Human GPR84 W145C in pcDps	This paper	N/A
HA-/FLAG-tagged Human GPR84 V149A in pcDps	This paper	N/A
HA-/FLAG-tagged Human GPR84 G190W in pcDps	This paper	N/A
HA-/FLAG-tagged Human GPR84 V227A in pcDps	This paper	N/A
HA-/FLAG-tagged Human GPR84 T264Ain pcDps	This paper	N/A
HA-/FLAG-tagged Human GPR84 S280N in pcDps	This paper	N/A
HA-/FLAG-tagged Human GPR84 K281N in pcDps	This paper	N/A
HA-/FLAG-tagged Human GPR84 P292T in pcDps	This paper	N/A
HA-/FLAG-tagged Human GPR84 A294V in pcDps	This paper	N/A
HA-/FLAG-tagged Human GPR84 S312L in pcDps	This paper	N/A
HA-/FLAG-tagged Human GPR84 A329P in pcDps	This paper	N/A
HA-/FLAG-tagged Human GPR84 A356V in pcDps	This paper	N/A
HA-/FLAG-tagged Human GPR84 Y370H in pcDps	This paper	N/A
HA-/FLAG-tagged Human GPR84 R375H in pcDps	This paper	N/A
HA-/FLAG-tagged Human GPR84 R378H in pcDps	This paper	N/A
HA-/FLAG-tagged Human GPR84 Y381N in pcDps	This paper	N/A
HA-/FLAG-tagged Human GPR84 P389H in pcDps	This paper	N/A
pcDps	(Okayama and Berg, 1983)	N/A

**Software and algorithms**		

BioEdit	(Hall, 1999)	https://bioedit.software.informer.com/7.2/RRID: SCR_007361
Biorender	BioRender.com	https://app.biorender.com/
Mega X	(Kumar et al., 2018)	https://www.megasoftware.net/
RELAX	(Wertheim et al., 2015)	https://www.datamonkey.org/relax
aBSREL	(Smith et al., 2015)	https://www.datamonkey.org/absrel
FEL	(Kosakovsky Pond and Frost, 2005)	https://www.datamonkey.org/fel
DNASTAR Lasergene	DNASTAR Lasergene	https://www.dnastar.com/software/lasergene/
GraphPad Prism	GraphPad Software	https://www.graphpad.com/scientific-software/prism/
SYBYL-X 2.0	Certara	https://www.certara.com/sybyl-x-software/
CorelDraw	Corel	https://www.coreldraw.com/

### Resource availability

#### Lead contact

Further information and requests for resources and reagents should be directed to and will be fulfilled by the lead contact, Claudia Stäubert (claudia.staeubert@medizin.uni-leipzig.de).

#### Materials availability

All unique plasmids generated in this study are available from the Lead contact.

### Experimental model and subject details

#### Cell line

CHO-K1 (ATCC Cat# CCL-61) cells were grown in Dulbecco’s Modified Eagle Medium: Nutrient Mixture F-12 (DMEM/F12) supplemented with 10% fetal bovine serum (FBS), 100 U/mL penicillin and 100 μg/mL streptomycin. The cells were maintained at 37°C in a humidified 5% CO_2_ incubator.

#### Chemical compounds studied in this article

3-Hydroxydecanoic acid (PubChem CID: 26612); Decanoic acid (PubChem CID: 2969); *cis*-2-Decenoic acid (PubChem CID: 5356596); *trans*-2-Decenoic acid (PubChem CID: 5282724).

### Method details

#### GPR84 ortholog identification, alignments and evolutionary analyses

Through extensive NCBI database mining and sequence assembly, followed by analysis, manual proof-reading and trimming, we created one alignment containing 114 vertebrate GPR84 nucleotide sequences (excluding mammals, except human GPR84) and one alignment with 112 mammalian GPR84 nucleotide sequences using the ClustalW algorithm in BioEdit (Hall, 1999) (Figures S1 and S2). The evolutionary history was inferred by using the Maximum Likelihood method based on the General Time Reversible model (Nei and Kumar, 2000). Sequences of all cloned orthologs were deposited in the database. All accession numbers are listed in Table S1. In MEGA X (Kumar et al., 2018), we computed a maximum likelihood tree with 1000 bootstrap replications for branch support for the vertebrate GPR84 orthologs. Using the alignment of all 112 mammalian species, we applied RELAX (Wertheim et al., 2015) to test for relaxed selection of the *Chiroptera* branch, which is made available as a web-based application on datamonkey.org (Weaver et al., 2018) (Figure S2). The adaptive branch-site random effects likelihood (aBSREL) was used to test for episodic diversifying selection within the *Carnivore* GPR84 tree (Shen et al., 2010; Smith et al., 2015) (Figure S5, Table S8). Fixed Effects Likelihood (FEL) analyses, which infer non-synonymous (dN) and synonymous (dS) site-specific substitution rates, were conducted to test for positively selected sites in panda and polar bear GPR84 in comparison to all other *Carnivora* (Kosakovsky Pond and Frost, 2005) (Figure S5, Table S9).

#### Cloning of GPR84 orthologs and generation of human GPR84 variants

Genomic DNA samples were prepared from tissues of selected mammals (sources listed in Table S11) as previously described (Peters et al., 2019). All primers used are listed in Table S12. PCR reactions were performed under variable annealing and elongation conditions. A standard PCR reaction (50 μL) contained genomic DNA (50 ng) with primers (500 nM each), Q5 Reaction Buffer (1×), dNTPs (200 μM), and Q5 High-Fidelity DNA Polymerase (1 U; NEB). The reactions were initiated with denaturation at 98°C for 30 s, followed by 30 cycles of denaturation at 98°C for 10 s, annealing at 58°C for 30 s, and elongation at 72°C for 1 min. A final extension step was performed at 72°C for 10 min.

The full-length mammalian GPR84 orthologs were epitope-tagged with an *N*-terminal hemagglutinin (HA) epitope (YPYDVPDYA) and a *C*-terminal FLAG tag (DYKDDDDK) by a PCR-based overlapping fragment approach to allow immunological detection and subsequently inserted into the mammalian expression vector pcDps (Okayama and Berg, 1983).

Primers for site-directed mutagenesis of human GPR84 were designed using PrimerX. The Quick Change PCR was performed using Phusion (Thermo Fisher) polymerase, followed by digestion with DpnI overnight and transformation into *Escherichia coli* DH5α (NEB).

The identity of all constructs and the correctness of all PCR-derived sequences were confirmed by sequencing (Seqlab). Newly obtained sequences were deposited at NCBI, accession numbers listed in Table S1.

#### Plasmid transfection and functional assays

CHO-K1 cells were split into 75 cm^2^-cell culture flasks (2.5 × 10^6^ cells/flask) and transfected with a total amount of 6 μg of plasmid the following day as previously described (Peters et al., 2020). For transient plasmid transfection, Lipofectamine 2000 (Thermo Fisher Scientific) was used.

#### Enzyme-linked immunosorbent assay (ELISA)

An indirect cellular ELISA was used to estimate cell surface expression of *N*-terminal HA-tagged receptor constructs and total receptor expression of full-length HA/FLAG double-tagged GPR84 constructs was assessed using a “sandwich ELISA”. One day after transfection, CHO-K1 cells were seeded into 48-well plates (8 × 10^4^ cells/well, cell surface expression) or 6-well plates (3 × 10^5^ cells/well, total expression). For cell surface expression analyses, the cells were fixed the following day, blocked with DMEM supplemented with 10% FBS for 1h at 37°C, and subsequently incubated with anti-HA-peroxidase-labeled high-affinity rat monoclonal antibody (Sigma Aldrich). Excess unbound antibody was removed by extensive washing and thereafter H_2_O_2_ and *ο*-phenylenediamine (2.5 mM each in 0.1 M phosphate-citrate buffer, pH 5.0) were added. The enzyme reaction was stopped by adding 1 M H_2_SO_4_ containing 0.05 M Na_2_SO_4_ and absorption was measured at 492 nm and 620 nm. For determination of total expression levels, transfected cells were harvested and membrane preparations were solubilized in lysis buffer (10 mM Tris/HCl, pH 7.4, 150 mM NaCl, 1 mM DTT, 1 mM EDTA, 1% deoxycholate, 1% Nonidet P-40, 0.2 mM PMSF, 10 μg/mL aprotinin) overnight. Microtiter plates were coated with a monoclonal antibody directed against the C-terminal FLAG tag (10 mg/mL in 0.05 M borate buffer; Sigma-Aldrich). After incubation with the solubilized membranes, bound full-length GPR84 proteins were detected with the peroxidase-labeled anti-HA antibody as described above (Sigma Aldrich).

#### Agonist stimulation and ALPHAScreen cAMP assay

For the ALPHAScreen cAMP assays, cells were seeded in 96-well plates one day after transfection (2 × 10^4^ CHO-K1 cells/well), 6 h past seeding the medium was changed to serum-free (SF) and 16 h later the assay was performed. The medium was removed from the plates and 50 μL HBSS/HEPES with 1 mM 3-isobutyl-1-methylxanthine (IBMX) was added for 5 min at room temperature. Cells were stimulated for 15min at 37°C with various agonist concentrations in HBSS/HEPES, containing 1 mM IBMX and 2 μM forskolin. Reactions were stopped on ice. Cells were lysed in 20 μL lysis buffer containing 1 mM IBMX and 5 μL of lysate from each well were transferred to a 384-well plate. According to the manufacturer’s protocol, the cAMP content of cell extracts was determined by a non-radioactive cAMP accumulation assay based on the ALPHAScreen technology (Perkin Elmer). GraphPad Prism version 8 was used for data analysis (GraphPad Software 8.4.3).

#### Structural modeling of GPR84

To obtain structural models of particular GPR84 orthologs (human, panda bear, polar bear) either in the inactive or active state conformation, we used the already determined dopamine D3 receptor structure (D3R, inactive, PDB ID: 3PBL(Chien et al., 2010)) and the D3R/G_i_ complex (PDB ID: 7cmv, (Xu et al., 2021)) as structural templates. The advantages to use these templates are 1. The same receptor as a template for both activity state-related conformations, which improves the reliability of comparisons between derived models; 2. Specific sequence features between GPR84 and D3R are highly similar (e.g., prolines in TMH2, see http://www.ssfa-7tmr.de (Worth et al., 2017)); 3. As GPR84, D3R is coupled to G_i_ and 4. D3R like GPR84 is not a peptide-ligand receptor. Receptors activated by peptides can have specific structural features related to the binding of larger peptides or hormone ligands (Heyder et al., 2021), which are likely absent in GPCRs with non-peptide ligands like GPR84.

The homology modeling protocol was similar to the already described approach (Bräunig et al., 2018). In brief, the templates were modified by deleting their bound ligands and any further additional proteins for protein optimization attributed to an increased expression and stability for structure determination. The sequences of the GPR84 orthologs and the template GPCRs were compared, and specifically, the loop length (extra- (EL) and intracellular loops (IL)) were manually adjusted by deletion (e.g., in IL2) or addition (e.g. EL2) of amino acids. In both templates, the complete IL3 was missing and is therefore also absent in the GPR84 models. IL3 of GPR84 is extremely long with ∼100 amino acids. The sequences of the optimized D3R templates (with a fixed and unmodified G_i_ bound at the active state D3R) were then substituted by corresponding GPR84 sequences, resulting in rough homology models of GPR84 orthologs to be modeled. The *N*-terminal amino acids S10-V22 (numbering based on human GPR84) were added manually, because of the missing template for the *N* terminus. These models were generated with the software SYBYL-X 2.0 (Certara, NJ, US) and optimized by energy minimization with the Amber99 force field until converging at a termination gradient of 0.05 kcal/(mol∗Å) under constrained backbone atoms. Sidechain orientations were optimized by a 2 ns molecular dynamics simulation (MD) with constraint backbone atoms. The receptor models (inactive) and the human GPR84/G_i_ complex were energetically minimized without any constraint.

Finally, we validated the reliability and comparability of our models concerning known insights from recent structure-function studies published by other GPR84 research groups (Nikaido et al., 2015; Mahmud et al., 2017). Our models agree with aspects of the GPR84 model by Al Mahmud et al. (Mahmud et al., 2017), specifically regarding the conformation and localization of the EL2, which subsequently determines the role of R172 as an essential key player for binding of diverse agonists, like e.g., C10 or embelin. The agonist embelin can be docked into our human GPR84/G_i_ complex model (Figure 3B) at R172 as a main-interaction partner. The resulting docking pose fulfills several criteria potentially associated with the agonistic activity of such ligands. These include contacts to the known activation-related “toggle switch” region (Visiers et al., 2002) with an aromatic amino acid at position 6.48 in TMH6 (superscripted numbers according to the unifying Ballesteros & Weinstein numbering for rhodopsin-like GPCRs (Ballesteros and Weinstein, 1995).

#### Minor allele frequencies analyses of the GenomeAsia 100 K database

Whole-genome data of individuals from various parts of Asia was obtained from GenomeAsia 100K (https://genomeasia100k.org), (GenomeAsia100K Consortium, 2019) in the form of a variant calling file (VCF). The VCF was subsequently converted into the PLINK.bed format using the--vcf command in PLINK v1.9 (Purcell et al., 2007). The family IDs of the individuals were obtained from the GenomeAsia100K consortium (GenomeAsia100K Consortium, 2019). Cluster-stratified Minor Allele Frequencies (MAF) defined by the family IDs were estimated using the--freq command in PLINK v1.9 with--family flag (Table S10).

Populations with higher Identity by descent (IBD) sharing for a given SNP were considered to be an honest proxy to assess whether that SNP is under natural selection for a given population, considering that natural selection promotes longer IBD segments. IBD sharing was calculated using the Pi-Hat statistic (Proportion IBD: P(IBD = 2) + 0.5∗P(IBD = 1)) implemented in PLINK v.1.9 using--genome command. Only the highest Pi-Hat value of 1 was employed for identifying the population(s) with high IBD sharing. The number of pairs sharing the highest Pi-Hat values (Pi-Hat = 1) in relation to the total number of pairwise comparisons for various populations present in the GenomeAsia data was calculated.

#### Calculation of amino frequencies at conserved motif positions

The Ballesteros & Weinstein residue table for human class A (Rhodopsin) GPCRs (excluding olfactory receptors) was downloaded from the GPCRdb (Isberg et al., 2017) and used to calculate the frequency of each amino acid at particular positions of interest.

### Quantification and statistical analysis

All experiments were performed at least three times, as indicated in the figure and table legends, and, unless otherwise indicated, they are presented as mean ± the standard error of the mean (SEM). EC_50_ values were determined in GraphPad Prism by fitting dose-dependence responses to a three-parameter sigmoidal curve (assuming a Hill slope of 1). Statistical analyses were performed using GraphPad Prism. As stated in Table S6 and Table S7, data were analyzed using unpaired two-tailed t-tests (p values with p ≤ 0.05 were considered statistically significant).

## Data Availability

All sequences of mammalian GPR84 orthologs functionally tested in the present study were deposited in the NCBI GenBank database (accession numbers are listed in Table S1). This article analyzes existing, publicly available data. These accession numbers are listed in Table S1. This article does not report original code. Any additional information required to reanalyze the data reported in this article is available from the lead contact on request.

## Supplemental information

Document S1. Figures S1–S7 and Tables S2, S3, S6, S8, and S11

Table S1. NCBI database accession numbers and sequence description of vertebrate GPR84 orthologs, related to Figures 1 and 2

Table S4. Conservation analyses of rhodopsin-like GPCRs, related to Figure 3

Table S5. Rhodopsin-like GPCR variants, related to Figure 3

Table S7. Summary of Emax, logEC50 and EC50 values of decanoic acid, 3-hydroxydecaonic acid, cis-2-decenoic acid, and trans-2-decenoic acid for all mammalian GPR84 orthologs, related to Figures 4 and 5

Table S9. FEL results detecting pervasive positive/diversifying in bears, related to Figure 4

Table S10. Naturally occurring mutations in human GPR84, related to Figure 6

Table S12. Primers used for GPR84 ortholog amplification, sequencing and introduction of epitope tags are listed as well as quick-change primer to introduce mutations at specific positions of human GPR84, related to STAR methods
