# Supplementary material for: Evolutionary analyses reveal immune cell receptor GPR84 as a conserved receptor for bacteria-derived molecules
Source: iScience. 2022 Sep 6;25(10):105087. doi: 10.1016/j.isci.2022.105087 (PMC9508565; doi:10.1016/j.isci.2022.105087)
Supplement: Document S1. Figures S1–S7 and Tables S2, S3, S6, S8, and S11 [file mmc1.pdf]

iScience, Volume *25*

Supplemental information

Evolutionary analyses reveal immune cell receptor GPR84 as a conserved receptor for bacteria-derived molecules

Amadeus Samuel Schulze, Gunnar Kleinau, Rosanna Krakowsky, David Rochmann, Ranajit Das, Catherine L. Worth, Petra Krumbholz, Patrick Scheerer, and Claudia Stäubert

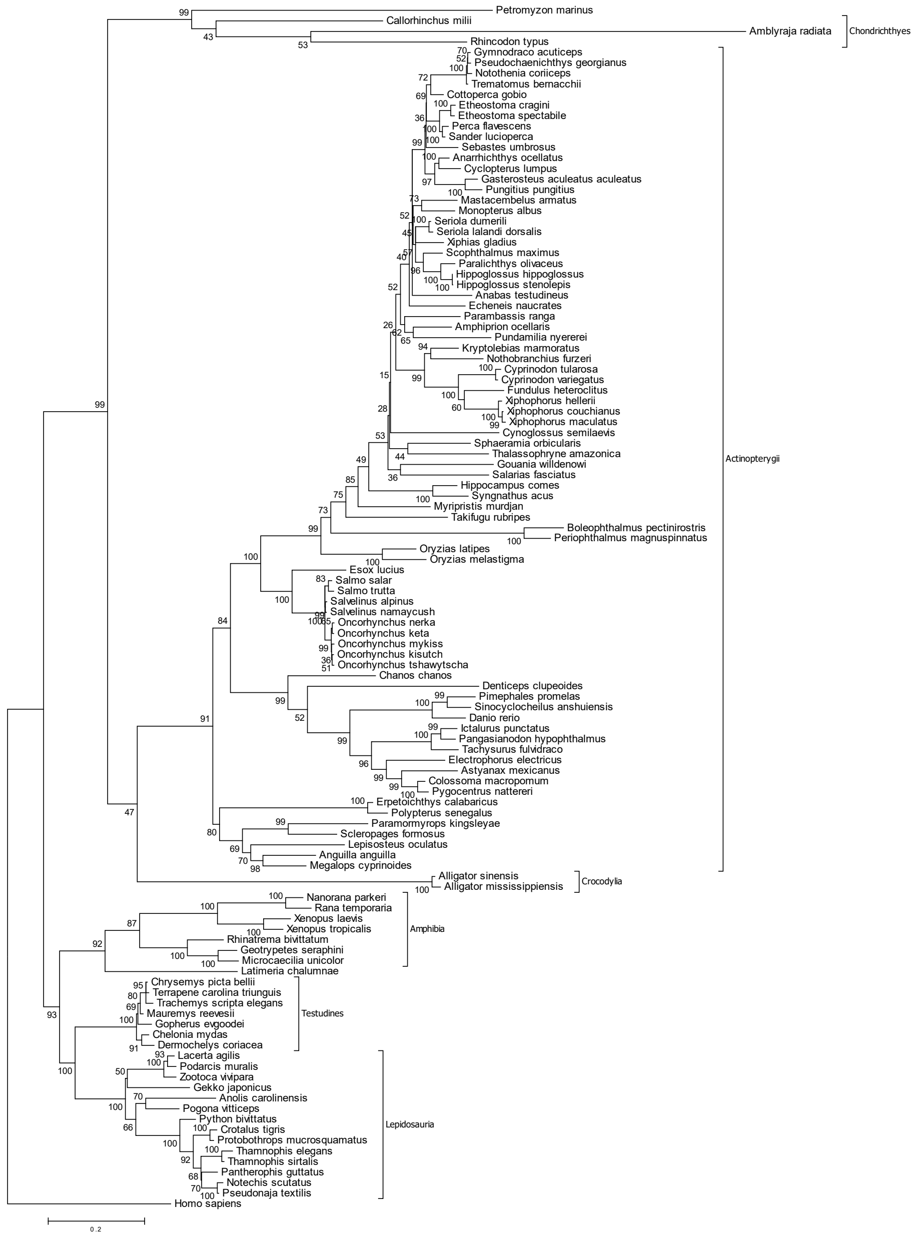

Figure S1 Molecular Phylogenetic analysis by Maximum Likelihood method of 114 vertebrate GPR84 orthologs.

The evolutionary history was inferred by using the Maximum Likelihood method based on the General Time Reversible model (Nei and Kumar, 2000). The tree with the highest log likelihood (-42936.4582) is shown. The percentage of trees in which the associated taxa clustered together is shown next to the branches. Initial tree(s) for the heuristic search were obtained automatically by applying Neighbor-Join and BioNJ algorithms to a matrix of pairwise distances estimated using the Maximum Composite Likelihood (MCL) approach, and then selecting the topology with a superior log likelihood value. A discrete Gamma distribution was used to model evolutionary rate differences among sites (5 categories (+G, parameter = 1.3167)). The rate variation model allowed for some sites to be evolutionarily invariable ([+I], 15.3272 % sites). The tree is drawn to scale, with branch lengths measured in the number of substitutions per site. The analysis involved 114 nucleotide sequences. Codon positions included were 1st+2nd+3rd+Noncoding. All positions with less than 95 % site coverage were eliminated. That is, fewer than 5 % alignment gaps, missing data, and ambiguous bases were allowed at any position. There were a total of 993 positions in the final dataset. Evolutionary analyses were conducted in MEGA X (Kumar et al., 2018). Related to Figure 1

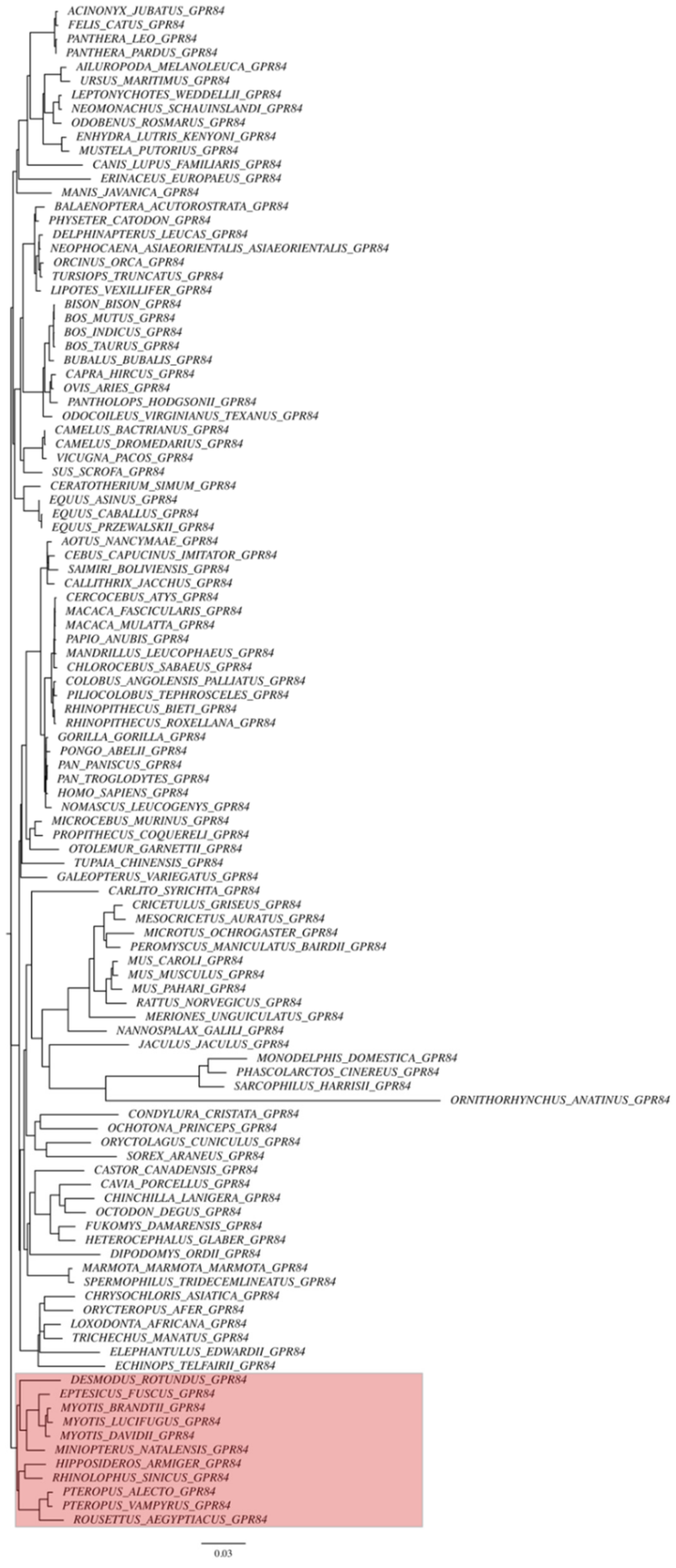

Figure S2 Phylogenetic tree of 112 mammalian GPR84 orthologs to test for relaxation of selective constraint in chiropteran GPR84.

Relaxed selection strength on the branch of bat GPR84 orthologs was confirmed by the RELAX hypothesis testing framework (Wertheim et al., 2015; Weaver et al., 2018). The phylogenetic tree represents all 112 mammalian GPR84 orthologs included in this analysis. All *Chiroptera* were selected as test branches (marked in red) and the remaining 101 orthologs as reference branches. The selection intensity parameter k = 0.07 was significantly lower than 1 (P < .001), indicating a relaxed selection strength on the GPR84 gene of bats. Related to Figure 1

A

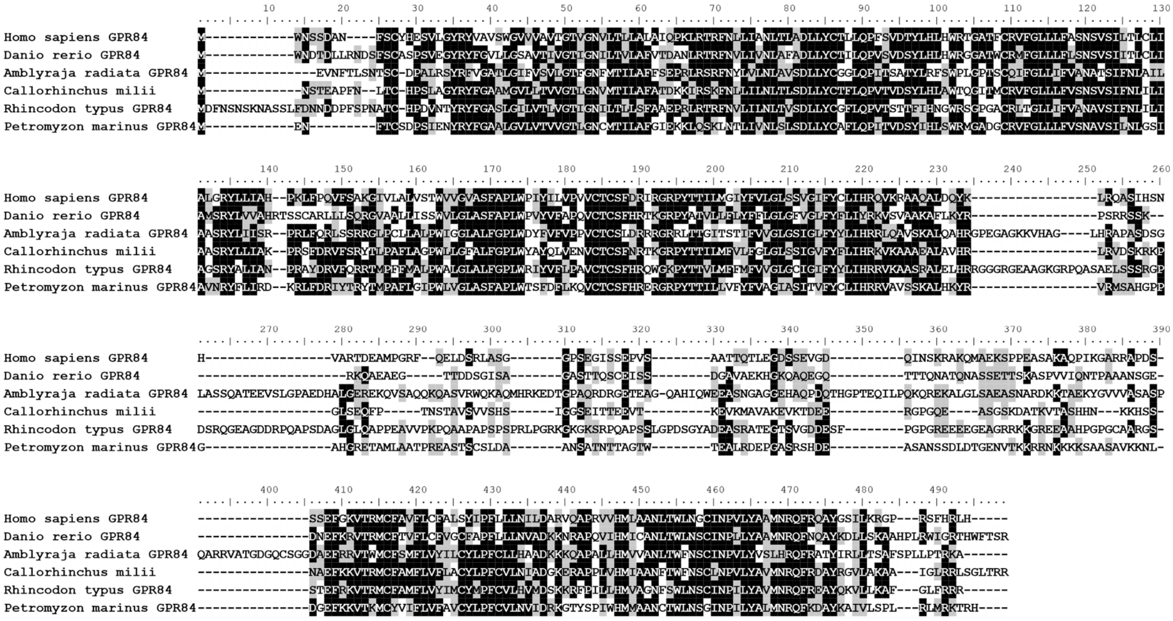

B

sequence identity (%)	*Homo sapiens*	*Danio rerio*	*Amblyraja radiata*	*Callorhinchus milii*	*Rhincodon typus*	*Petromyzon marinus*
Homo sapiens	ID	48	31	47	35	41
Danio rerio	48	ID	32	44	36	40
Amblyraja radiata	31	32	ID	35	38	34
Callorhinchus milii	47	44	35	ID	44	46
Rhincodon typus	35	36	38	44	ID	38
Petromyzon marinus	41	40	34	46	38	ID

Figure S3. Comparison of GPR84 protein sequences of sea lamprey and cartilaginous fishes to human and zebrafish.
(**A**) Protein alignment of human and zebrafish GPR84 versus whale shark (*Rhincodon typus*), Australian whale shark (*Callorhinchus milii*), thorny skate (*Amblyraja radiata*) and sea lamprey (*Petromyzon marinus*). GPR84 ortholog data was retrieved from database mining (NCBI accession numbers collected in Table S1(Hall, 1999)). (**B**) % sequence identity was determined from the protein alignment shown in (**A**) using *BioEdit*. Related to Figure 1

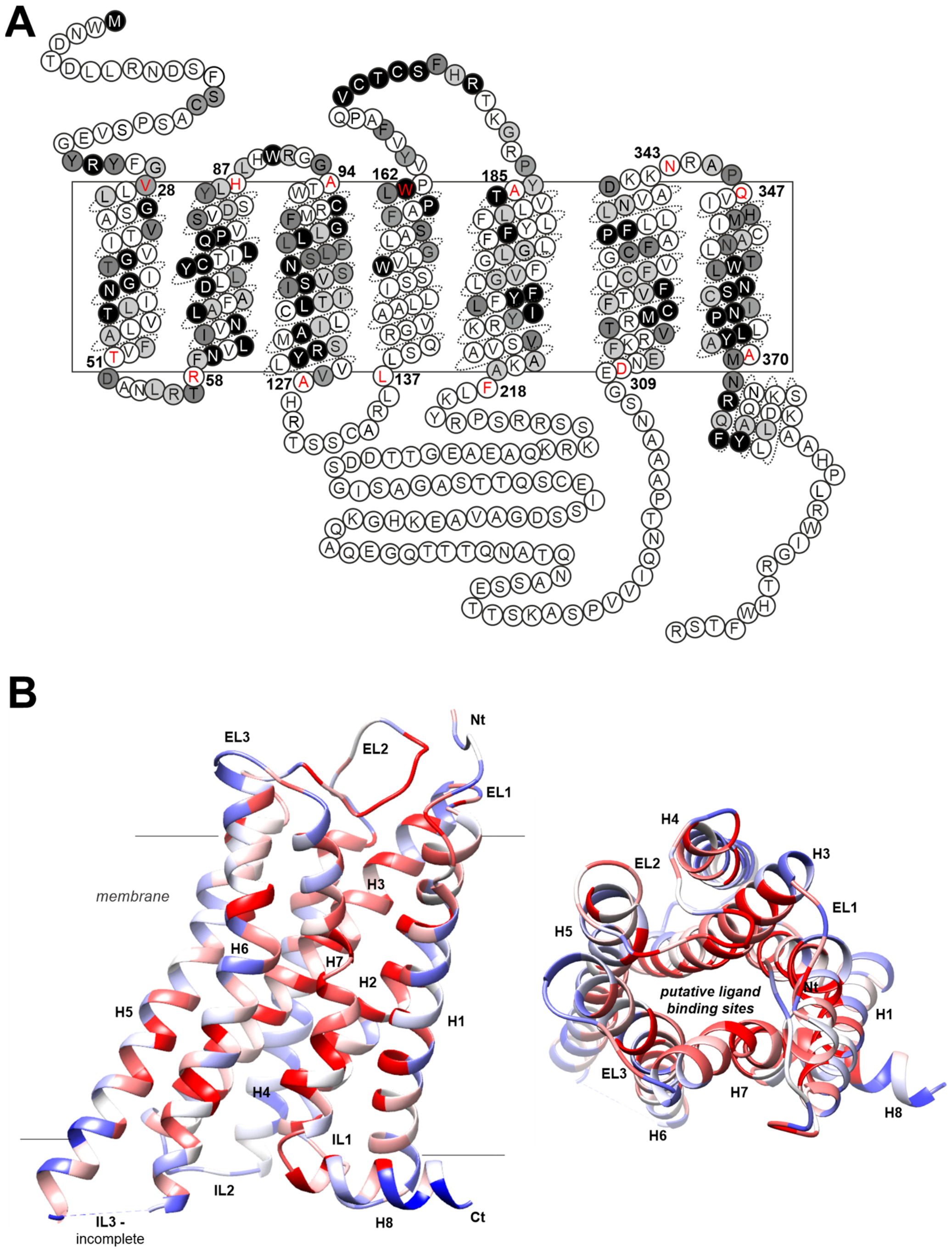

Figure S4. Snake plot and model of GPR84 visualizing conservation of GPR84 among 113 non-mammalian vertebrates.

(**A**) Zebrafish GPR84 snake-plot highlighting positional conservation among 113 non-mammalian GPR84 vertebrate orthologs. Positions that are 100 % conserved are shown in black, positions at which 2 to 3 different amino acids are tolerated are shown in grey, positions with higher variation rates are shown in white. Indicated in red are the predicted transmembrane helix border positions. (**B**) Conservation of the 113 non-mammalian vertebrate species visualized at a GPR84 3-dimensional homology model (left–side view, right - top view). The software *Chimera* (Pettersen et al., 2021) was used. Red indicates high conservation of amino acid positions, blue low conservation. The inner core of the transmembrane helical bundle including cover-like arranged parts of the EL2 is most conserved, while membrane orientated side chains are less-conserved. TMH3 shows the highest conservation in the amino acid composition. IL – intracellular loop, EL – extracellular loop, Ct – C terminus, Nt – N terminus, H: Helix. Related to Figure 2

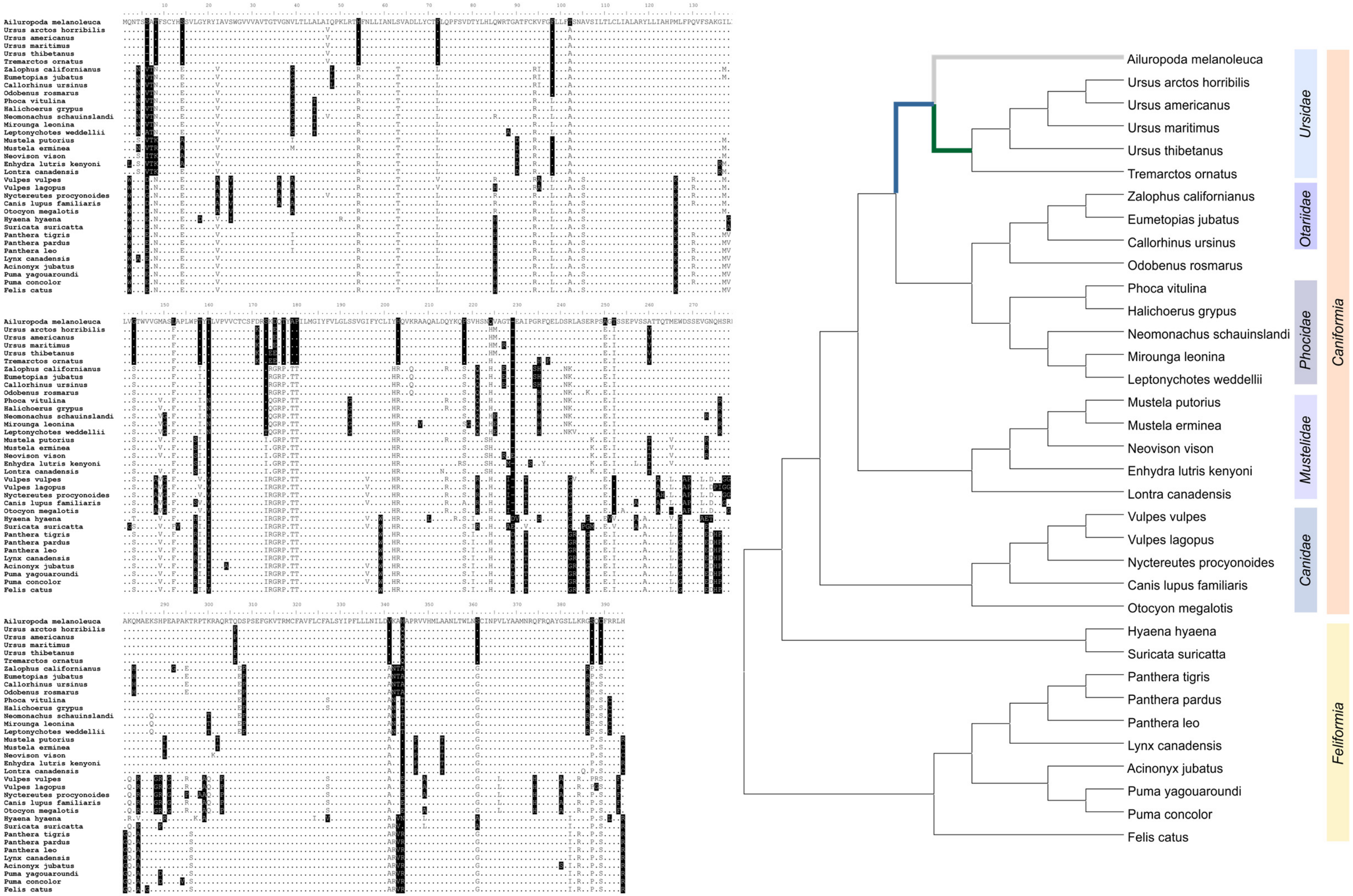

Figure S5. *Carnivora* GPR84 alignment and tree for testing of selection.

Protein alignment of *Carnivora* GPR84 highlighting positional conservation and differences and corresponding arbitrary phylogenetic tree used in selection analyses. Evidence for episodic diversifying selection was identified for the node to *Ursidae* (blue) ($p = 0.0179$) and node to *Ursidae* excluding *Ailuropoda melaneuca* (green, $p = 0.0013$) using aBSREL (Table S8) (Smith et al., 2015). Related to Figure 4

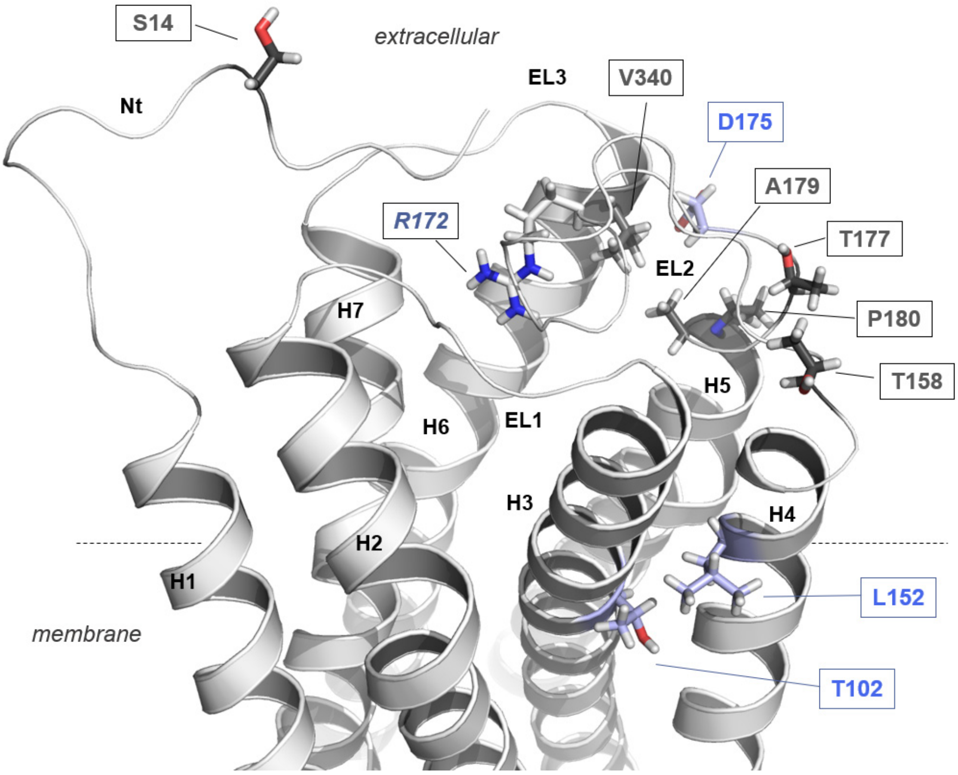

Figure S6. Panda bear GPR84 model with specific amino acids highlighted as compared to other mammals and in particular *Carnivora*. According to the results of our analyses summarized in Table S9, eight positions (gray sticks, two positions are not included in the model) in the *Ursidae* are specific compared to other mammalian GPR84 sequences. Five of these residues specifically cluster in the N- and *C*-terminal parts of EL2, at the transitions to TMH4 and TMH5. Val^{340} in TMH6 additionally participates in this spatial clustering and interacts directly with Ala^{179}. This cluster is close to Arg^{172}, which is discussed to be directly involved in ligand binding (see Figure 3B). In conclusion, bear-specific residues are related to the EL2 and likely modify properties of this receptor part (adjustment, dynamics), which is involved in ligand binding. Residues Thr^{102}, Leu^{152} and Asp^{175} are specific for Panda bear (blue sticks). Thr^{102} and Leu^{152} are in direct contact at the interface between TMH3 and TMH4 (corresponding evolutionary substitution). Asp^{175} participates in the bear-specific cluster at the *C*-terminal EL2, close to the ligand-binding sensitive Arg^{172}. Therefore, from a spatial perspective, these three panda-specific variants should significantly differentiate the panda GPR84 from other vertebrate receptor orthologs. Related to Figure 4

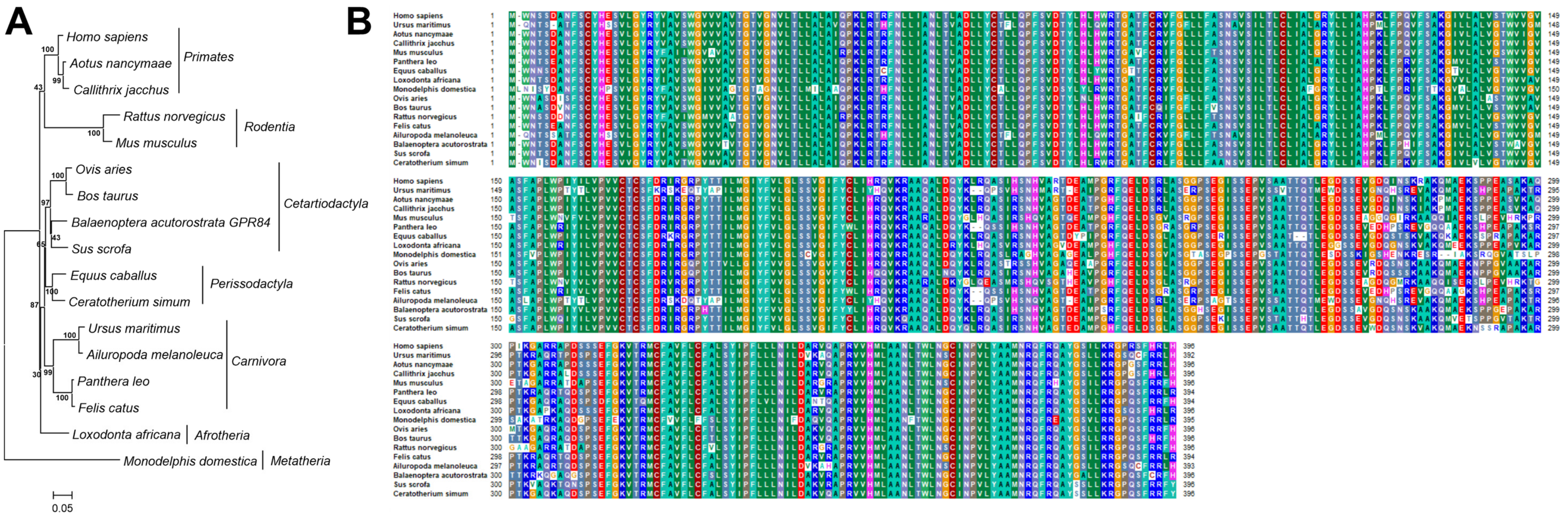

Figure S7. Phylogenetic tree and protein alignment of functionally characterized GPR84 orthologs.

(**A**) Phylogenetic tree of all functionally characterized species. Bootstrap values are shown in percent. The evolutionary history was inferred by using the Maximum Likelihood method and Tamura 3-parameter model. The tree with the highest log likelihood (-6815.40) is shown. The percentage of trees in which the associated taxa clustered together is shown next to the branches. Initial tree(s) for the heuristic search were automatically obtained by applying Neighbor-Joining and BioNJ algorithms to a matrix of pairwise distances estimated using the Maximum Composite Likelihood (MCL) approach, and then selecting the topology with superior log likelihood value. A discrete Gamma distribution was used to model evolutionary rate differences among sites (5 categories (+G, parameter = 0.5392)). The tree is drawn to scale, with branch lengths measured in the number of substitutions per site. This analysis involved 17 nucleotide sequences. There were a total of 1194 positions in the final dataset. Evolutionary analyses were conducted in MEGA X (Kumar et al., 2018). (**B**) Termini, transmembrane helices and loops of the functionally characterized receptor orthologs are marked in the alignment according to the GPR84 homology model designed within this study. Related to Figure 4 and 5

Table S2

Degree of conservation at specific positions and occurrence of alternative amino acids in rhodopsin-like GPCRs. Specific corresponding positions in rhodopsin-like GPCRs (defining the unifying Ballesteros & Weinstein numbering scheme (Ballesteros and Weinstein, 1995)) were analyzed concerning the degree of conservation. In addition, residues comprising highly conserved motifs (e.g. the *DRY* motif in TMH3) are included. The exact degree of conservation (sequence identity), alternative amino acids (with the highest and low occurrence) and examples of receptors with different residues are presented, the GPR84 is highlighted bold. For the calculations 287 human rhodopsin-like GPCRs were used (~380 olfactory class A GPCRs were excluded). In addition, the degree of conservation is provided for mammalian and non-mammalian GPR84 orthologs. Related to Figure 3

**Type of amino acid (one-letter code)**	**Ballesteros & Weinstein numbering**	**Structural localization**	**Conservation among class A GPCRs (%)**	**Variations with >4% conservation**	**Other variations with <4% conservation**	**GPCR examples**	**Conservation in mammalian GPR84** (set of 101 sequences)	**Conservation in non-mammalian vertebrate GPR84** (set of 113 sequences)
**N**	1.50	TMH1	**98.25**	-	S, G	GPR171 (S)	N (101)	N (113)
**D**	2.50	TMH2	**91.96**	-	A, E, G, H, L, N, Q, S, Y	GnRH1 (L), NTS2 (G), LGRs4-6 (N)	D (101)	D (113)
**C**	3.25	TMH3	**88.03**	-	A, D, F, I, H, K, N, Q, P, S, R, T, W, V	MC1-5Rs (D), S1P1R (W)	C (101)	C (113)
**D**	3.49		**64.34**	E (21.3 %)	A, G, F, H, L, N, Q, S, R, T, V	CCK1R (E), NTS1R (E), PAR2R (Q), **GPR84 (G)**	G (89)<br>A (12, all *Carnivora*)	G (11 ray-finned fish)<br>S (101)
**R**	3.50		**94.76**	-	C, G, F, H, K, L, Q, T, Y	LGR6 (C), RXFP1R (K), GPR148 (T), DP1R (C)	R (101)	R (113)
**Y**	3.51		**66.43**	C (10. 5%), F (10.5 %)	A, M, G, H, L, S, R, W	C5a1R (F), Y4R (H), OX1R (W), C3aR (C)	Y (100)<br>F (1, *Trichechus manatus*)	Y (113)
**W**	4.50	TMH4	**95.8**	-	C, F, L, S, V, Y	EP1R (A), LGRs4-6 (F, A, L)	W (101)	W (113)
**P**	5.50	TMH5	**79.02**	-	A, C, F, L, S, V, Y, M	MC1-5Rs (M), TSHR (A), S1P1R (L), CB1R (L), P2Y12 (N), **GPR84 (G)**	G (101)	G (111)<br>S (1, *Lepisosteus oculatus*)<br>A (2, *Erpetoichthys calabaricus, Polypterus senegalus*)
**C**	6.47	TMH6	**70.63**	S (10.1 %)	A, F, M, L, T, W	M1-5R (T), C5a1R (F), CCR1-8 (F), FFA4R (M), **GPR84 (S)**	S (101)	**S (21)**<br>**C (92)**
**W**	6.48		**67.83**	F (16.4 %)	A, C, E, G, I, M, L, N, Q, S, R, T, V, Y	OX1R (Y), PAR1-4 (F), TSHR (M), LGR5 (N), **GPR84 (Y)**	Y (101)	**Y (38)**<br>**F (75)**
**P**	6.50		**99.3**	-	Y, V	GPR148 (V)	P (101)	P (113)
**N**	7.49	TMH7	**71.93**	D (20 %)	I, H, K, L, S, T, V	MC1-5R (D), PAR1-4 (D), GPR62 (H), P2Y11 (H)	N (101)	N (113)
**P**	7.50		**94.04**	-	A, C, F, M, L, T, V	MT1 (A), PKR1 (T), GPR148 (M)	P (99)<br>T (1, *Elephantulus edwardii*)<br>gap (Echinops telfairi)	P (113)
**Y**	7.53		**89.12**	F (4.2 %)	C, I, M, L, P, T, H, V	ETAR (L), FFA1R (T), P2Y6 (F), GPR162 (I)	Y (100)<br>F (1, *Ochotona princeps*)<br>gap (Echinops telfairi)	Y (113)
**F**	8.50	Helix 8	**65.4**	L (8.1 %), V (5.7 %), Y (5.7 %)	A, C, D, G, I, M, Q, R	5HT2AR, GHSR, GnRH1, PAR3, FFA1R, S1P1R	F (101)	F (113)

Table S3

Conservation and variations of prototypical amino acid motifs in rhodopsin-like GPCRs. Four conserved amino acid motifs located in different transmembrane helices are commonly found in rhodopsin-like GPCRs. The most highly conserved residues (bold) have critical structural and/or functional roles, although amino acid variations do occur at these positions. Related to Figure 3

**Commonly found rhodopsin-like motifs**	**Structural localization**	**Conservation**	**Alternative motifs**	**Conservation**	**Total conservation**
$_{3.49}$*D**R**Y*	TMH3	**48 %**	*E**R**Y*	7.3 %	**69.97 %**
			*D**R**F*	6.97 %	
			*E**R**C*	4.2 %	
			*D**R**C*	3.5 %	
$_{5.58}$***Y*(7x)*K(R)***	TMH5	**20.2 %**	***Y*(7x)*L***	7.7 %	**40.1 %**
			Y*(7x)W*	6.97 %	
			Y*(7x)Q*	5.23 %	
$_{6.47}$*CWx**P***	TMH6	**47.4 %**	*CFx**P***	14.3 %	**73.18 %**
			*SWx**P***	6.6 %	
			*LWx**P***	4.88 %	
$_{7.49}$*N**P**xxY(5,6x)F*	TMH7-Helix H8	**46.3 %**	*D**P**xxY(5,6x)F*	8.3 %	**57.7 %**
			*N**P**xxY(5,6x)Y*	3.1 %	

Table S6

Cell surface and total receptor expression, basal activity of all functionally characterized HA- and FLAG-tagged GPR84 orthologs. Expression data was determined by ELISA. Specific optical density (OD) readings are given as percentage of the HA-tagged human GPR84. The OD value of the HA- and FLAG-tagged human GPR84 (set 100 %) was 0.153 ± 0.015 in cell surface and 0.166 ± 0.033 in total expression measurements. The non-specific OD value (empty vector) was 0.026 ± 0.003 cell surface and 0.022 ± 0.005 in total expression measurements. Data are given as mean ± SEM of ≥ 3 independent experiments carried out in triplicates. CHO-K1 cells were transfected with GPR84-ortholog constructs and cAMP inhibition assays in presence of 2 μM forskolin were performed. The basal activity was determined by cAMP inhibition assays and is given as % of unstimulated empty vector (mean ± SEM) of ≥ 6 independent experiments carried out in triplicates. The basal cAMP level of mock-transfected CHO-K1 stimulated with 2 μM forskolin (control) was 3.65 ± 0.25 nmol/well. Unpaired two-tailed t-test comparing mammalian GPR84 orthologs to human GPR84 were used to determine P-values (* P ≤ 0.05, ** P ≤ 0.01, *** P ≤ 0.001). Related to Figure 4

	**Cell surface expression**	**Total expression**	**Basal activity**
	**% of human receptor construct**		**% of unstimulated empty vector**
Human	100 (6)	100 (6)	47 ± 5.0
Azara‘s night monkey	181 ± 10 (5)***	109 ± 12 (4)	46 ± 5.2
Common marmoset	147 ± 14 (3)**	107 ± 9.4 (3)	46 ± 3.6
Brown rat	310 ± 34 (6)***	218 ± 22 (6)***	44 ± 4.0
House mouse	106 ± 14 (3)	88 ± 8.4 (3)	43 ± 4.0
Sheep	160 ± 29 (5)*	58 ± 6.3 (4)***	42 ± 3.0
Cattle	139 ± 11 (5)**	113 ± 8.7 (5)	44 ± 3.6
Pig	138 ± 22 (3)*	143 ± 29 (3)	51 ±4.2
Minke whale	76 ± 8.8 (3)**	29 ± 3.8 (3)***	67 ± 4.4***
Horse	70 ± 7.4 (4)***	120 ± 6.6 (3)**	40 ± 2.3
White rhinoceros	111 ± 9.3 (3)	78 ± 17 (3)	53 ± 4.6
Polar bear	384 ± 16 (3)***	94 ± 5.7 (3)	63 ± 5.0**
Giant panda	356 ± 35 (4)***	137 ± 13 (4)**	69 ± 3.9***
Lion	160 ± 14 (3)***	108 ± 6.3 (3)	43 ± 2.5
Cat	161 ± 17 (5)**	118 ± 19 (5)*	53 ± 3.9
African elephant	*128 ± 12 (5)**	*101 ± 14 (4)*	*40 ± 5.6*
Gray short-tailed opossum	*187 ± 13 (6)****	*32 ± 2.6 (4)****	*65 ± 5.6***

Table S8

Among all *Carnivora* (Figure S5), aBSREL found evidence of episodic diversifying selection on the node shared by polar bear and panda (Likelihood Ratio Test $p \leq 0.05$, after correcting for multiple testing). Test for selection relaxation ($k = 0.72$) using RELAX was not significant ($p = 0.230$, LR = 1.44) for the node *Ursidae* (Wertheim et al., 2015). However, for the node to *Ursidae w/o Ailuropoda melaneuca* RELAX analyses revealed that the test for selection intensification ($k = 2.32$) was significant ($p = 0.012$, LR = 6.25). Related to Figure 4

Name	LRT	Test p-value	Uncorrected p-value	ω distribution over sites
Node to *Ursidae* (Figure S5, blue)	10.3143	0.0179	0.0020	ω1 = 0.691 (99 %)
				ω2 = 100000 (1.2 %)
Node to *Ursidae w/o Ailuropoda melaneuca* (Figure S5, green)	15.6607	0.0013	0.0001	ω1 = 0.560 (99 %)
				ω2 = 100000 (0.61 %)

Table S11

Sources of genomic DNA used for mammalian GPR84 amplification. Related to STAR methods

Species latin	Species english	Source
Ailuropoda melanoleuca	Giant panda	Dr C. Pitra, IZW, Berlin, Germany
Aotus azarae	Azara’s night monkey	Dr T. Haaf, MPI Molecular Genetics Berlin, German
Balaenoptera acutorostrata scammoni	Minke whale	Dr P. J. Palsboll, University of Wales, U.K.
Bos taurus	Cattle	Dr C. Pitra, IZW, Berlin, Germany
Callithrix jacchus	Common marmoset	Dr T. Haaf, MPI Molecular Genetics Berlin, German
Ceratotherium simium	White rhinoceros	Dr C. Pitra, IZW, Berlin, Germany
Equus przewalskii	Horse	Dr C. Pitra, IZW, Berlin, Germany
Felis catus	Cat	Dr C. Pitra, IZW, Berlin, Germany
Homo sapiens	Human	Dr C. Pitra, IZW, Berlin, Germany
Loxodonta africana	African elephant	Dr C. Pitra, IZW, Berlin, Germany
Monodelphis domestica	Gray short-tailed opossum	Dr C. Pitra, IZW, Berlin, Germany
Mus musculus	House mouse	Dr C. Pitra, IZW, Berlin, Germany
Ovis aries	Sheep	Dr C. Pitra, IZW, Berlin, Germany
Panthera leo	Lion	Dr C. Pitra, IZW, Berlin, Germany
Rattus norvegicus	Brown rat	Dr C. Pitra, IZW, Berlin, Germany
Sus scrofa	Pig	Dr C. Pitra, IZW, Berlin, Germany
Ursus maritimus	Polar bear	Dr C. Pitra, IZW, Berlin, Germany
